# FACT plays a major role in histone dynamics affecting *VSG* expression site control in *Trypanosoma brucei*

**DOI:** 10.1111/mmi.12812

**Published:** 2014-10-22

**Authors:** Viola Denninger, Gloria Rudenko

**Affiliations:** Division of Cell and Molecular Biology, Sir Alexander Fleming Building, Imperial College LondonSouth Kensington, London, SW7 2AZ, UK

## Abstract

Chromatin remodelling is involved in the transcriptional regulation of the RNA polymerase I transcribed variant surface glycoprotein (VSG) expression sites (ESs) of *Trypanosoma brucei*. We show that the *T. brucei* FACT complex contains the Pob3 and Spt16 subunits, and plays a key role in ES silencing. We see an inverse correlation between transcription and condensed chromatin, whereby FACT knockdown results in ES derepression and more open chromatin around silent ES promoters. Derepressed ESs show increased sensitivity to micrococcal nuclease (MNase) digestion, and a decrease in histones at silent ES promoters but not telomeres. In contrast, FACT knockdown results in more histones at the active ES, correlated with transcription shut-down. ES promoters are derepressed in cells stalled at the G2/M cell cycle stage after knockdown of FACT, but not in G2/M cells stalled after knockdown of cyclin 6. This argues that the observed ES derepression is a direct consequence of histone chaperone activity by FACT at the G2/M cell cycle stage which could affect transcription elongation, rather than an indirect consequence of a cell cycle checkpoint. These experiments highlight the role of the FACT complex in cell cycle-specific chromatin remodelling within VSG ESs.

## Introduction

In eukaryotes gene expression is typically coordinated by complicated protein networks, which extend far beyond the scope of the basic transcription machinery. Packaging of DNA into different types of chromatin plays a key role in determining and maintaining the activation state of genes (Cairns, 2009; Margueron and Reinberg, 2010; van Steensel, 2011). The eukaryotic protozoan parasite *Trypanosoma brucei* has very few transcription factors and very little transcriptional control (Clayton, 2002; Berriman *et al*., 2005; Ivens *et al*., 2005). Most *T. brucei* genes are present in very extensive polycistronic transcription units, which are constitutively transcribed by RNA polymerase II (Pol II) (Berriman *et al*., 2005). As the regulation of gene expression is predominantly post-transcriptional in this organism (Kramer, 2012), this makes the role of chromatin structure and remodelling an intriguing issue.

The bloodstream-form trypanosome expresses an essential protective VSG coat (Sheader *et al*., 2005) from a single copy *VSG* gene located in one of about 15 telomeric ES transcription units (Berriman *et al*., 2002; Hertz-Fowler *et al*., 2008). During the course of a chronic infection, trypanosomes can switch the active *VSG* either through switching to a new ES, or through DNA rearrangements replacing the *VSG* at the active ES telomere (reviewed in Taylor and Rudenko, 2006; Horn and McCulloch, 2010; Glover *et al*., 2013; Horn, 2014). Unusually, the active ES is transcribed by RNA polymerase I (Pol I) (Gunzl *et al*., 2003), which in eukaryotes normally exclusively transcribes rDNA. This unusual and highly regulated Pol I-mediated mono-allelic transcription of ESs in *T. brucei* is key for antigenic variation to work (Borst, 2002).

It is now clear that chromatin remodelling plays a key role in ES control (Rudenko, 2010; Glover *et al*., 2013). ESs have different chromatin structures depending on their activation state, whereby the active Pol I-transcribed ES is very extensively depleted of nucleosomes (Figueiredo and Cross, 2010; Stanne and Rudenko, 2010). In addition, a broad range of chromatin proteins, remodellers and histone chaperones have now been shown to be involved in ES control. Knockdown of chromatin proteins and remodellers (ISWI, NLP, RAP1), histone chaperones (FACT, ASF1, CAF-1b), histone modifiers (DAC3, DOT1B), or histones H1 or H3 results in derepression of silent ES promoters or changes in VSG switching (Hughes *et al*., 2007; Figueiredo *et al*., 2008; Yang *et al*., 2009; Denninger *et al*., 2010; Wang *et al*., 2010; Narayanan *et al*., 2011; Alsford and Horn, 2012; Povelones *et al*., 2012; Pena *et al*., 2014).

In addition, there appears to be a link between chromosome replication and segregation and ES silencing, as knockdown of the Origin Recognition Complex (ORC), MCM-BP or the cohesin subunit TbSCC1 affects telomeric silencing of *VSG*s (Landeira *et al*., 2009; Tiengwe *et al*., 2012; Benmerzouga *et al*., 2013; Kim *et al*., 2013). Nuclear architecture also plays a role in the maintenance of silenced chromatin domains, as knockdown of the lamin-like protein NUP1 results in perturbation of *VSG* silencing (DuBois *et al*., 2012). Although most research has concentrated on chromatin proteins and remodellers silencing *VSG*s, chromatin proteins also play a role in ensuring high levels of expression of the active *VSG* ES. For example, the HMG-box containing chromatin protein TDP1 is key for transcription of the active ES (Narayanan and Rudenko, 2013). TDP appears to coat the transcriptionally active ES in a mutually exclusive fashion to the linker histone H1, which is enriched on silent ESs (Povelones *et al*., 2012; Pena *et al*., 2014). Possibly TDP1 protects DNA that is stripped of nucleosomes, and helps maintain the open chromatin state necessary for high levels of Pol I transcription.

The FACT (Facilitates Chromatin Transcription) complex is a histone chaperone which alters chromatin structure by actively disassembling and reassembling nucleosomes, and has been shown to play a key role in ES control (Belotserkovskaya *et al*., 2003; Denninger *et al*., 2010). FACT has been implicated in multiple processes which rely on functional chromatin structure like DNA replication, chromosome segregation, transcription initiation and elongation (Lejeune *et al*., 2007; Formosa, 2012). FACT plays a role in nucleosome reorganization by binding to the nucleosome, converting it from a stable to an unstable form and vice versa (McCullough *et al*., 2011; Winkler and Luger, 2011). During this nucleosome destabilization, although there is disruption of contacts between histone H2A–H2B dimers, H3–H4 tetramers and DNA, the ratio of histone H2A–H2B to H3–H4 dimers does not appear to change (Xin *et al*., 2009; McCullough *et al*., 2011).

Disruption of chromatin remodellers and histone chaperones in *T. brucei* can produce a cell cycle arrest coupled with ES derepression (Alsford and Horn, 2012). Knockdown of the *T. brucei* FACT large subunit Spt16 results in an accumulation of up to 40% cells at the G2/M cell cycle stage (Denninger *et al*., 2010). This G2/M arrest has also been observed after the knockdown of histone H3 and to some extent the histone chaperone CAF-1b, although in this latter case derepression in S phase was also observed (Alsford and Horn, 2012). One hypothesis has been that the observed ES derepression seen in cells stalled after knockdown of FACT (Denninger *et al*., 2010), is a direct consequence of this G2/M cell cycle arrest, and that all ES promoters are normally transiently derepressed at this cell cycle stage. We investigated this issue through an analysis of the *T. brucei* FACT complex, including its role in chromatin structure as influenced by histone distribution.

After depletion of FACT, we find a reduction in histones around silent ES promoters which become transcriptionally derepressed. In addition, there is an increase in histones along the active ES which becomes silenced, as has been shown earlier in Denninger *et al*. (2010). This inverse correlation between histone deposition and ES transcription parallels observed changes in chromatin structure after FACT knockdown, as probed using micrococcal nuclease (MNase). Arresting cells at G2/M by knockdown of cyclin 6 did not result in ES derepression. This argues that the observed ES derepression after FACT knockdown is due to a reduction in histone chaperone activity resulting in changes in chromatin structure, rather than a consequence of ES derepression triggered by a G2/M cell cycle checkpoint. Cell cycle-specific chromatin remodelling by FACT, particularly around the ES promoters, therefore plays an important role in ES control. As well as its role in ES regulation, we show the importance of the FACT complex in global histone distribution and transcription in *T. brucei*.

## Results

### The *T. brucei* FACT complex is composed of the Spt16 and Pob3 subunits

Eukaryotic FACT complexes typically contain two or three subunits. In *Saccharomyces cerevisiae*, the large subunit Spt16 forms a stable complex with the histone chaperone Pob3 and interacts with the small HMG-box protein Nhp6 (Wittmeyer *et al*., 1999; Formosa *et al*., 2001). In mammals, Pob3 and Nhp6 appear to be replaced by a single protein SSRP1 (structure-specific recognition protein 1), which contains the histone chaperone domain of Pob3 as well as the DNA-binding domain of Nhp6 (Orphanides *et al*., 1999). We used tandem affinity purification in order to determine the composition of the *T. brucei* FACT complex. One allele of the Spt16 large subunit was tagged with a C-terminal PTP-epitope at the endogenous Spt16 locus in procyclic-form *T. brucei* (Schimanski *et al*., 2005). The second Spt16 allele could be knocked out without leading to a growth defect demonstrating that the tagged PTP-Spt16 allele was functional (data not shown).

**Figure 1:**
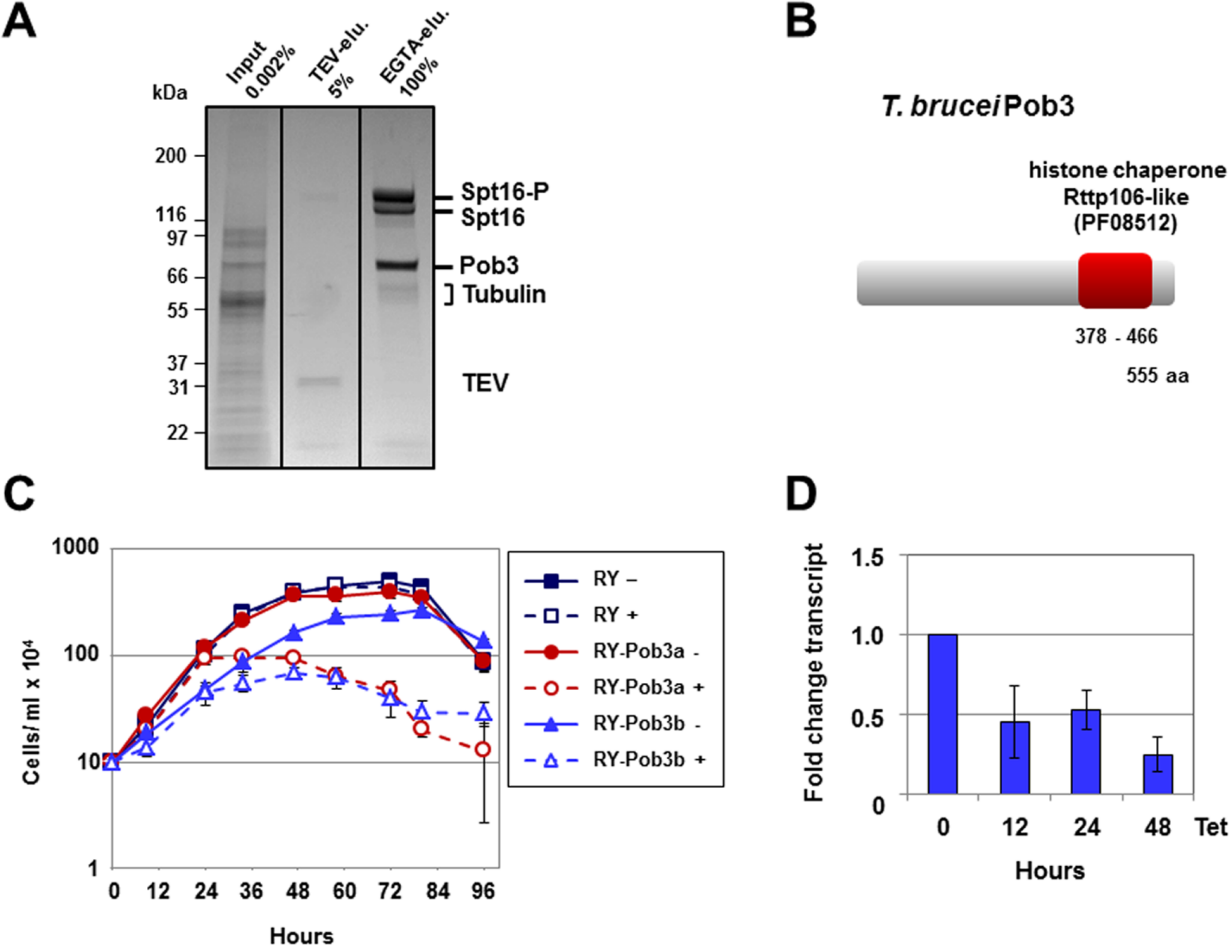
The *T. brucei* FACT complex consists of the Spt16 and Pob3 subunits.A. Affinity purification of the FACT complex using PTP-tagged Spt16 identifies the Pob3 subunit with an approximate size of 70 kiloDalton (kDa). A representative SDS-PAGE gel stained with Coomassie blue is shown. Total input (0.002% crude extract) in the first lane is compared with TEV-eluate (TEV-elu.) obtained after the first purification step and cleavage with the TEV-protease (5% total) (second lane). The final eluate after elution with EGTA (EGTA-elu.) is shown in the third lane. The major bands are: Spt16-P (Spt16 tagged with the protein C epitope), Spt16 (epitope cleaved off), and Pob3. The cleaved off TEV epitope and contaminating tubulin are also shown. Identity of the bands was established using mass spectrometry analysis. Size markers are indicated in kDa on the left.B. Protein motif analysis of *T. brucei* Pob3 (Accession No.: Tb927.10.14390) using the SMART program reveals a highly conserved histone chaperone Rtt106-like domain (*e*-value: 4.9e^−22^).C. Pob3 is essential in bloodstream-form *T. brucei*. The graph shows the cell density of the RY-Pob3a and RY-Pob3b cell lines in comparison with the parental *T. brucei* RY strain in the presence (+) or absence (−) of tetracycline to induce Pob3 RNAi. The mean of three measurements is plotted against time, with standard deviation indicated with error bars.D. Decrease in Pob3 transcript after induction of Pob3 RNAi in the *T. brucei* RY-Pob3a cell line with tetracycline (Tet). Knockdown was monitored via qPCR analysis, and plotted as fold change in transcript level over time. The mean of three independent experiments is shown with standard deviation indicated with error bars.

In order to purify Spt16 interacting proteins and determine the composition of the FACT complex, the lysate of about 2 × 10^10^ procyclic-form cells was purified over two subsequent immunoaffinity columns, before the Spt16 containing protein complex was eluted. The final EGTA eluate was loaded on to a 10% SDS-polyacrylamide gel and the bands subjected to mass spectrometry (Fig. 1A, Fig. S1). A single Spt16-interacting protein of about 70 kDa was found, which was the *T. brucei* orthologue of the small FACT subunit Pob3. Pob3 had earlier been identified in *T. brucei* using bioinformatic searches (Patrick *et al*., 2008). *T. brucei* Pob3 is a well-conserved protein of 555 amino acids containing a histone chaperone domain (Fig. 1B). Depletion of Pob3 in bloodstream-form *T. brucei* using tetracycline inducible RNAi resulted in a growth arrest comparable to that found after the induction of Spt16 RNAi (Fig. 1C and D) (Denninger *et al*., 2010).

Mass spectrometry did not identify an Nhp6 orthologue or other DNA-binding protein (Fig. S1). The FACT complex in *T. brucei* therefore only appears to be composed of the Spt16 and Pob3 subunits, although we cannot exclude the existence of other interaction partners which interact either transiently, or not strongly enough to survive the purification conditions. However even in yeast, Nhp6 is just a facultative partner of FACT, and can interact with multiple complexes (Formosa *et al*., 2001; Stillman, 2010).

**Figure 2:**
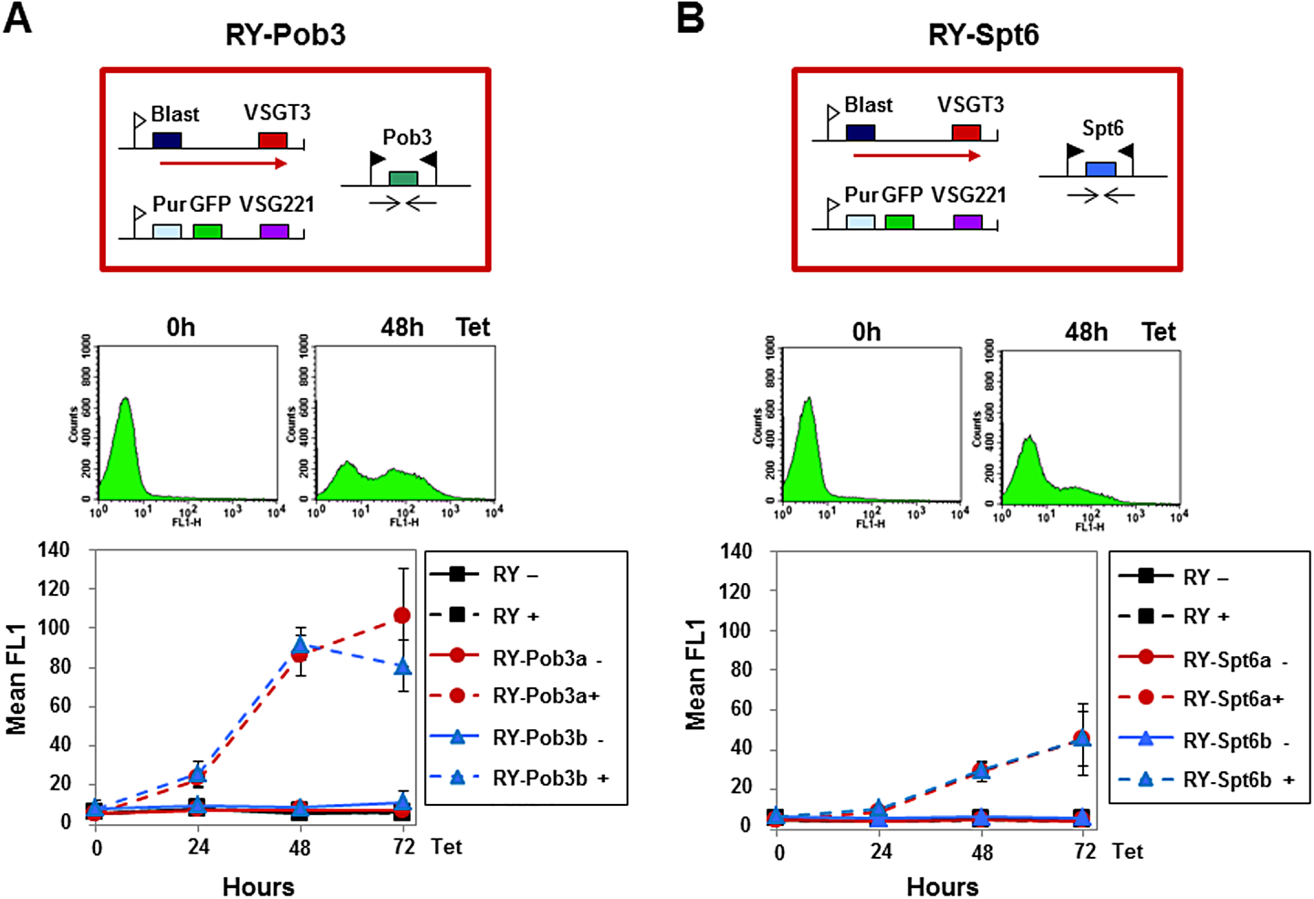
Derepression of silent *VSG* ESs after knockdown of the FACT small subunit Pob3.A. Significant ES derepression after depletion of Pob3. The schematic shows the *T. brucei* RY-Pob3 RNAi cell line with a blasticidin (Blast) resistance gene in the active *VSGT3* ES and a puromycin (Pur) resistance and GFP gene in the silent *VSG221* ES. The ES promoters are shown as white flags with transcription indicated with a red arrow. The Pob3-RNAi fragment is transcribed from two opposing T7-promoters (black flags). Below, flow cytometry traces show analysis of RY-Pob3 cells before or after the induction of Pob3-RNAi with tetracycline (Tet) for the time indicated in hours (h). Derepression of the silent *VSG221* ES promoter is monitored as an increase in green fluorescence in the FL1 channel (*x*-axis). The graph shows the mean fluorescence in the FL1 channel through time after induction of Pob3-RNAi in the RY-Pob3a and RY-Pob3b cell lines compared with the parental RY line. The mean of three independent experiments is plotted with the standard deviation indicated with error bars.B. There is no significant ES derepression after knockdown of the putative histone chaperone Spt6. Analysis of the *T. brucei* RY-Spt6a and R-Spt6b cell lines after the induction of Spt6 RNAi with tetracycline. Further this panel is as described in panel A.

In order to monitor the effect of Pob3 depletion on silencing of *VSG* expression sites (ES)s, Pob3 knockdown was performed in the *T. brucei* RY-T3 cell line, which has a *GFP* gene integrated immediately downstream of the silent *VSG221* ES promoter (Hughes *et al*., 2007). Flow cytometry revealed a 10-fold increase in green fluorescence after 48 h induction of Pob3 RNAi, indicating derepression of the silent *VSG221* ES (Fig. 2). This result is comparable to that observed after knockdown of the Pob3 FACT subunit partner Spt16 (Denninger *et al*., 2010).

### Knockdown of Spt6 leads to lethality but not ES derepression in bloodstream-form *T. brucei*

Spt6 is another putative histone chaperone, which has been reported to act in concert with FACT to facilitate RNA polymerase II (Pol II) transcription in yeast and mammalian cells (Bortvin and Winston, 1996; Kaplan *et al*., 2003; Belotserkovskaya and Reinberg, 2004). We performed bioinformatic searches of the *T. brucei* TriTryp sequence database using *S. cerevisiae* Spt6. We identified an Spt6 orthologue in the *T. brucei* 927 and *T. brucei* Lister 427 strains with scores of 3.2 e^−07^ and 4.1 e^−07^ respectively. Although Spt6 sequence identity is conserved over the length of the *T. brucei* Spt6 orthologue, we could only identify two of the conserved Spt6 domains (YqgF and SH2) (Close *et al*., 2011). Knockdown of Spt6 in bloodstream-form *T. brucei* resulted in a growth arrest as observed after knockdown of Spt16 or Pob3 (Fig. S2). However, in contrast to as observed after knockdown of these two FACT subunits, depletion of Spt6 in the *T. brucei* RY-T3 cell line resulted in only minor derepression of the silent *VSG221* ES (about fourfold after 48 h induction of Spt6 RNAi (Fig. 2B) (Denninger *et al*., 2010).

### Depletion of Spt16 results in an increase in mononucleosomes specifically downstream of silent ES promoters

**Figure 3:**
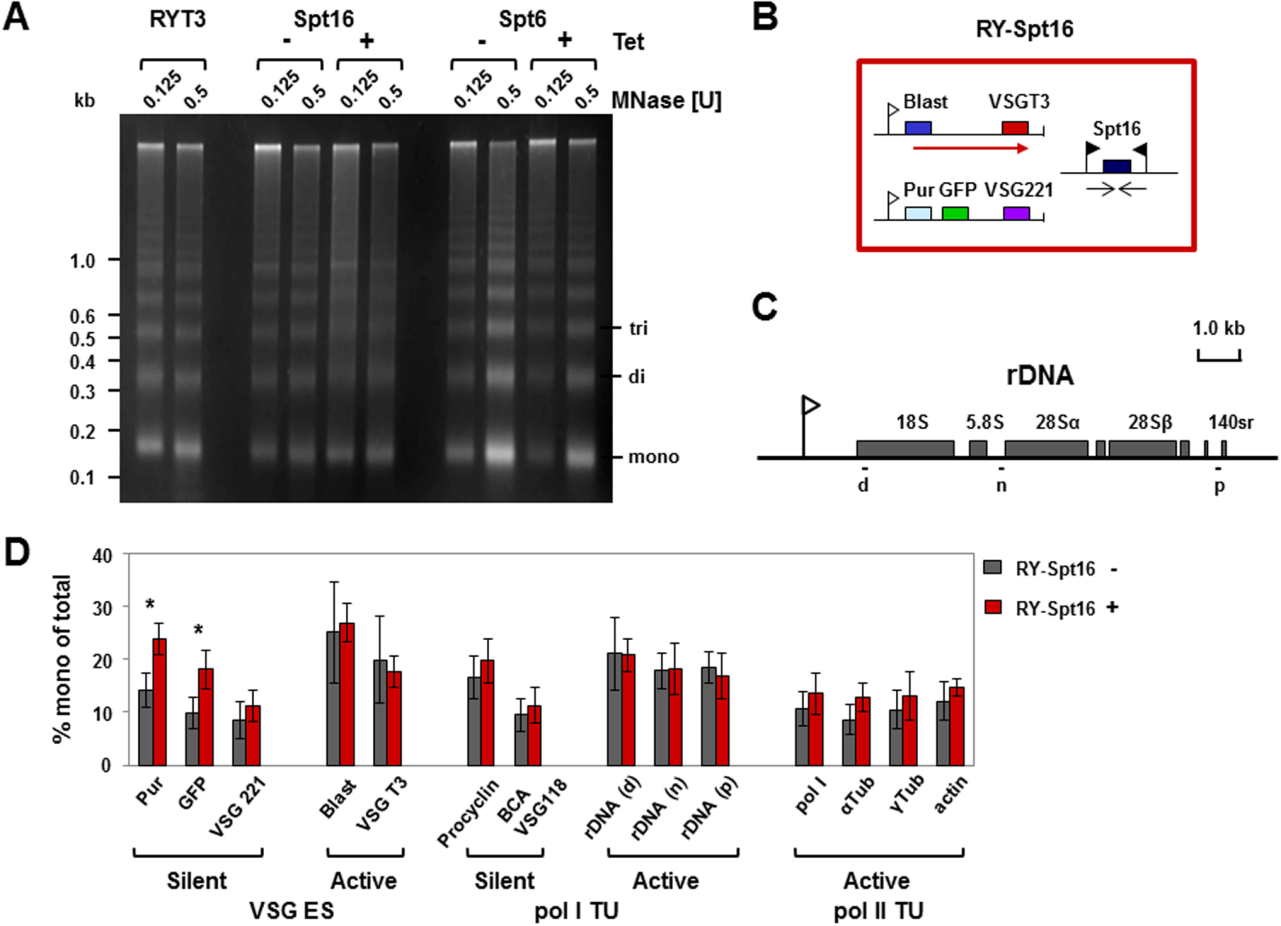
Depletion of the FACT large subunit Spt16 affects the general abundance and spacing of nucleosomes, and specifically results in more open chromatin structure at silent ES promoters.A. Analysis of chromatin isolated from bloodstream-form *T. brucei* after knockdown of Spt16 or Spt6 in comparison with the parental RYT3 strain. *T. brucei* RY-Spt16 or RY-Spt6 cells were grown for 24 h in the presence (+) or absence (−) of tetracycline (Tet) to induce either Spt16 or Spt6 RNAi. Chromatin from an equal number of cells was isolated and digested with 0.125 or 0.5 units (U) of micrococcal nuclease (MNase). Equal amounts of DNA were loaded on an ethidium bromide-stained gel. A representative experiment with size markers indicated in kilobases (kb) is shown.B. Schematic of the *T. brucei* RY-Spt16 cell line (large red box). The *VSGT3* ES is active (transcription indicated with a red arrow). A blasticidin (Blast) resistance gene is located downstream of the active *VSGT3* ES promoter (white flag), and puromycin (Pur) resistance and GFP genes are located downstream of the silent *VSG221* ES promoter. The Spt16 RNAi fragment is transcribed from two opposing tetracycline inducible T7 promoters (black flags).C. Schematic of a *T. brucei* rDNA transcription unit with the rRNA genes indicated with filled boxes and the rDNA promoter with a flag. Regions which were analysed by qPCR after MNase digestion are indicated with lettered black lines.D. Knockdown of Spt16 results in a significant increase in mononucleosomes (indicative of increased MNase sensitivity and a more open chromatin structure) downstream of the silent *VSG221* ES promoter. MNase digestion and nucleosome fractionation was performed according to Stanne and Rudenko (2010). The abundance of mononucleosomes at different genomic locations was determined by qPCR analysis, and plotted as the percentage of total input. The chromatin state of silent and active *VSG* ESs was compared in the presence (+) or absence (−) of tetracycline to knockdown Spt16. In addition, genes present in RNA polymerase I transcription units (Pol I TU) which are silent [procyclin or containing a basic copy array (BCA) *VSG*] or active (rDNA) were compared. Analysis of the active Pol II transcription units [RNA polymerase I large subunit (Pol I), α-tubulin, γ-tubulin and actin] is also shown. The results are the mean of three independent experiments, with the standard deviation indicated with error bars. Significance was determined by a Student's *t*-test (unpaired, two-tailed) with significance indicated with * (*P* = 0.01–0.05).

Histone chaperones actively disassemble and reassemble nucleosomes by binding histones and removing them, in contrast to chromatin remodellers like ISWI which slide nucleosomes to facilitate access to the DNA. We investigated how knockdown of the putative histone chaperones FACT or Spt6 affect chromatin structure using micrococcal nuclease (MNase) digestion of chromatin (Stanne and Rudenko, 2010). We digested chromatin with MNase after 24 h induction of Spt16 or Spt6 RNAi, and compared the digestion patterns with those obtained from uninduced cells as well as the parental *T. brucei* strain RY-T3. The *T. brucei* RY-T3 cell line has a blasticidin resistance gene downstream of the active *VSGT3* ES promoter, as well as a puromycin resistance and *GFP* gene downstream of the silent *VSG221* ES promoter (Hughes *et al*., 2007), enabling experimental distinction of the active *VSGT3* and the silent *VSG221* ES. In the parental RY-T3 cells or uninduced trypanosomes, distinct nucleosomal laddering is observed, with sharp bands indicating precise phasing of nucleosomes (Fig. 3A).

Depletion of the FACT large subunit Spt16 resulted in MNase chromatin digestion patterns changing from relatively discrete nucleosomal ladders to more diffuse laddering. This indicates that knockdown of Spt16 results in a general disruption of nucleosomal phasing. Genomic loci which are present in a relatively open chromatin state are preferentially digested by MNase into mononucleosomes (Figueiredo and Cross, 2010; Stanne and Rudenko, 2010). This open chromatin conformation can be due to a decrease in chromatin condensation, for example as was observed earlier after knockdown of the linker histone H1 (Povelones *et al*., 2012; Pena *et al*., 2014). Alternatively, open chromatin structure can also be a consequence of a reduction in the levels of core histones, which can be determined using chromatin immunoprecipitation (see below). After blocking Spt16 synthesis, no general accumulation of mononucleosomes was observed, indicating that Spt16 depletion primarily affects nucleosome spacing. In contrast to as seen with Spt16, knockdown of the putative histone chaperone Spt6 resulted in sharp nucleosomal laddering similar to as seen in the parental strain, indicating that nucleosomal phasing had not been affected.

In order to determine which genomic loci were particularly affected by Spt16 knockdown, we coupled the MNase digestion assay with nucleosome fractionation on sucrose density gradients as described in Stanne and Rudenko (2010) and Povelones *et al*. (2012). Mononucleosomal fractions of samples before or after depletion of Spt16 were pooled, the DNA isolated and subjected to quantitative PCR (qPCR). The relative presence of mononucleosomes within the investigated DNA loci was expressed as percentage of the total input (sum of all nucleosomal fractions) (Fig. 3D) (Povelones *et al*., 2012). In wild-type cells, the active *VSGT3* ES is present in a highly open chromatin state, and of the genomic loci tested, the most MNase-sensitive region with the highest abundance of mononucleosomes (20–25%) is found at the active *VSGT3* ES (Fig. 3B and D). These results are comparable to those obtained from the rDNA locus, which is also transcribed by Pol I (Fig. 3C and D) (Stanne and Rudenko, 2010). Interestingly, MNase digestion of genomic regions like the tubulin and actin loci, which are constitutively transcribed by Pol II, only resulted in 10 to 15% mononucleosomes. This is comparable to values observed within the silent *VSG22*1 ES. This indicates that areas constitutively transcribed by pol II have a far less open chromatin state than regions actively transcribed by Pol I, which are strikingly depleted of nucleosomes (Figueiredo and Cross, 2010; Stanne and Rudenko, 2010).

Induction of an Spt16 synthesis block resulted in a relative opening of the chromatin structure at the promoter of the silent *VSG221* ES, which contains the single copy puromycin resistance and *GFP* genes (Fig. 3B and D). Here, MNase digestion resulted in a statistically significant increase in the mononucleosomal fraction, with levels increased to those found at the active *VSGT3* ES (Table S1). This opening of the chromatin structure did not extend down to the silent telomeric *VSG221* gene itself. This agrees with data showing derepression of silent *VSG* ES promoters after knockdown of Spt16, but no increase in transcripts from the silent telomeric *VSG*s themselves (Denninger *et al*., 2010). We do not know if this increase in transcription from the silent ESs is due to an increase in rates of transcription initiation, or increased transcription elongation from stalled RNA polymerases (Kassem *et al*., 2014). Depletion of Spt16 did not result in a significant increase in mononucleosomes at either pol I or pol II transcribed areas or at non-transcribed loci. This argues that the FACT complex specifically plays a role in maintaining a relatively closed chromatin state at silent *VSG* ES promoters.

The results from separate nucleosomal fractions analysed individually were also plotted according to Povelones *et al*. (2012) which gave comparable results (Figs S3 and S4). We compared the levels of different nucleosome species (mono, di, di/tri, tri/tetra, greater than tetra) before and after depletion of Spt16, in order to analyse more global changes in nucleosome distribution. The relative percentage of each nucleosomal fraction within the sample was calculated, and the ratio (before and after induction of Spt16 RNAi) was plotted for each genomic locus respectively (Figs S3 and S4; Table S2). After knockdown of Spt16, the most striking increase in MNase sensitivity (increase in mononucleosomes) was observed around the silent VSG ES promoter.

### Knockdown of Spt16 leads to an increase in histones at active ESs, and a decrease in histones at silent ESs

**Figure 4:**
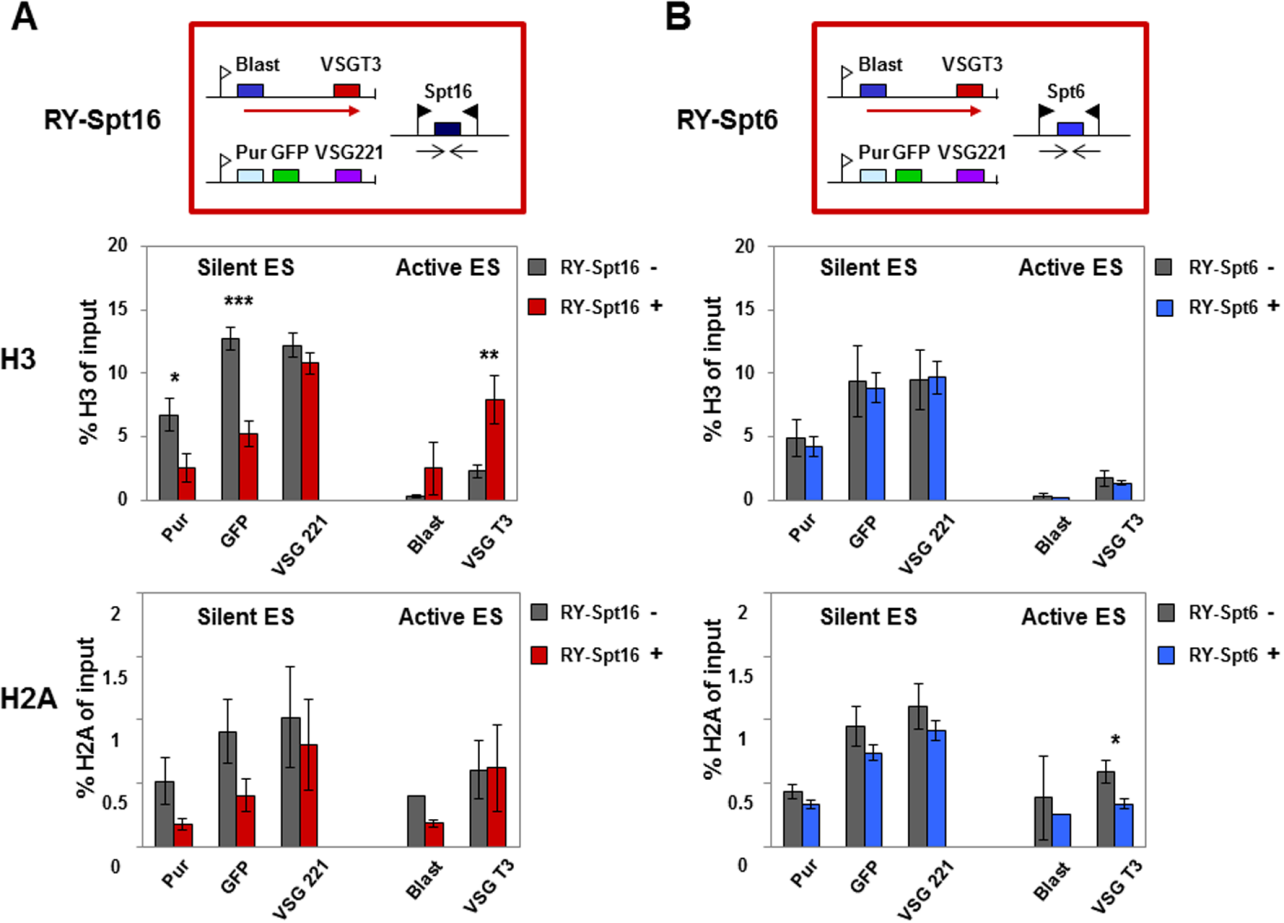
Knockdown of the FACT large subunit Spt16 results in histone H3 levels decreasing at silent ES promoters and increasing at the active ES.A. Changes in the abundance of histones H3 or H2A at the silent *VSG221* ES or active *VSGT3* ES in the presence (+) or absence (−) of RNAi against the FACT large subunit Spt16 for 24 h as determined by ChIP. The schematic shows the *T. brucei* RY-Spt16 cell line with the blasticidin (Blast) resistance gene inserted behind the promoter of the active *VSGT3* ES, and the puromycin (Pur) resistance and GFP genes integrated at the silent *VSG221* ES promoter as indicated in Fig. 2. The upper bar graph shows the abundance of histone H3 at the silent *VSG221* ES compared with the active *VSGT3* ES. In the lower bar graph comparable experiments show the abundance of histone H2A after knockdown of Spt16 with RNAi. Statistical significance of the results was determined with significant: * (*P* = 0.01–0.05); very significant: ** (*P* = 0.001–0.01) and extremely significant: *** (*P* < 0.001) indicated.B. Knockdown of the putative histone chaperone Spt6 does not lead to extensive changes in levels of histones H3 or H2A at ESs. This panel is as described in panel A, only Spt6 was analysed instead of Spt16.

In order to determine histone distribution after knockdown of the FACT Spt16 large subunit, we performed chromatin immunoprecipitation (ChIP) of histones H3 and H2A after 24 h knockdown of Spt16 or Spt6 respectively in the *T. brucei* RY-T3 cell line (Fig. 4; Tables S3 and S4). At the silent *VSG221* ES, knockdown of Spt16 lead to a statistically highly significant decrease in histone H3, in addition to a similar decrease in histone H2A (Fig. 4A). In the latter case, the lack of statistical significance was presumably due to lower affinity of the anti-H2A antibody, which resulted in lower amounts immunoprecipitated and slightly higher standard deviations.

In contrast, Spt16 knockdown resulted in a striking and statistically significant increase in the level of histone H3 at the active *VSGT3* ES (*P* = 0.001–0.01). Levels of histone H2A appeared to remain relatively comparable at the active ES. However, as much lower levels were immunoprecipitated and the results were not statistically relevant, we cannot draw strong conclusions from this result. This observed increase in levels of histone H3 (and presumably other histones) at the active ES, could result in changes to the chromatin structure which lead to decrease in the active *VSGT3* transcript as reported previously (Denninger *et al*., 2010). These alterations in histone H3 abundance were not simply a consequence of lethality of the Spt16 knockdown, as depletion of the essential protein Spt6 did not lead to striking changes in histone H3 (or H2A) at these loci (Fig. 4B; Tables S3 and S4).

Spt16 is found at the Pol I transcribed rDNA locus in *T. brucei* (Fig. S5A). In general there was a trend that levels of histone H3 increased at the rDNA locus after knockdown of Spt16 but not Spt6, although due to experimental variability this was only statistically significant using one primer pair (Fig. S6, Tables S3 and S4). In order to determine if increased histone levels were correlated with changes in transcription, we determined the abundance of three rRNA precursor transcripts (White *et al*., 1986) using qPCR (Fig. S6A). We found that some of the rRNA precursor transcripts decrease significantly over time after the induction of Spt16-RNAi (Fig. S6B, Table S5). Although the promoter proximal Precursor 1 transcript remained unaffected by Spt16 knockdown, there was a pronounced decrease in the downstream Precursor 2 and 3 transcripts. This could indicate decreased processivity of Pol I transcription. It has previously been observed that there is reduced transcript from the telomeric *VSG* at the active *VSG* ES after Spt16 knockdown (Denninger *et al*., 2010) compared with from the promoter proximal blasticidin gene (V. Denninger and G. Rudenko, unpubl. results). As FACT is thought to remove nucleosomes before the elongating RNA polymerase, reduced transcriptional processivity after its knockdown is to be expected. These data indicate that Pol I transcription is inversely correlated with histone abundance at both *VSG* ESs and rDNA loci, and that FACT plays a key role in controlling this genomic distribution of histones.

### Spt16 knockdown results in a decrease in histones at Pol II loci and strand switch regions

**Figure 5:**
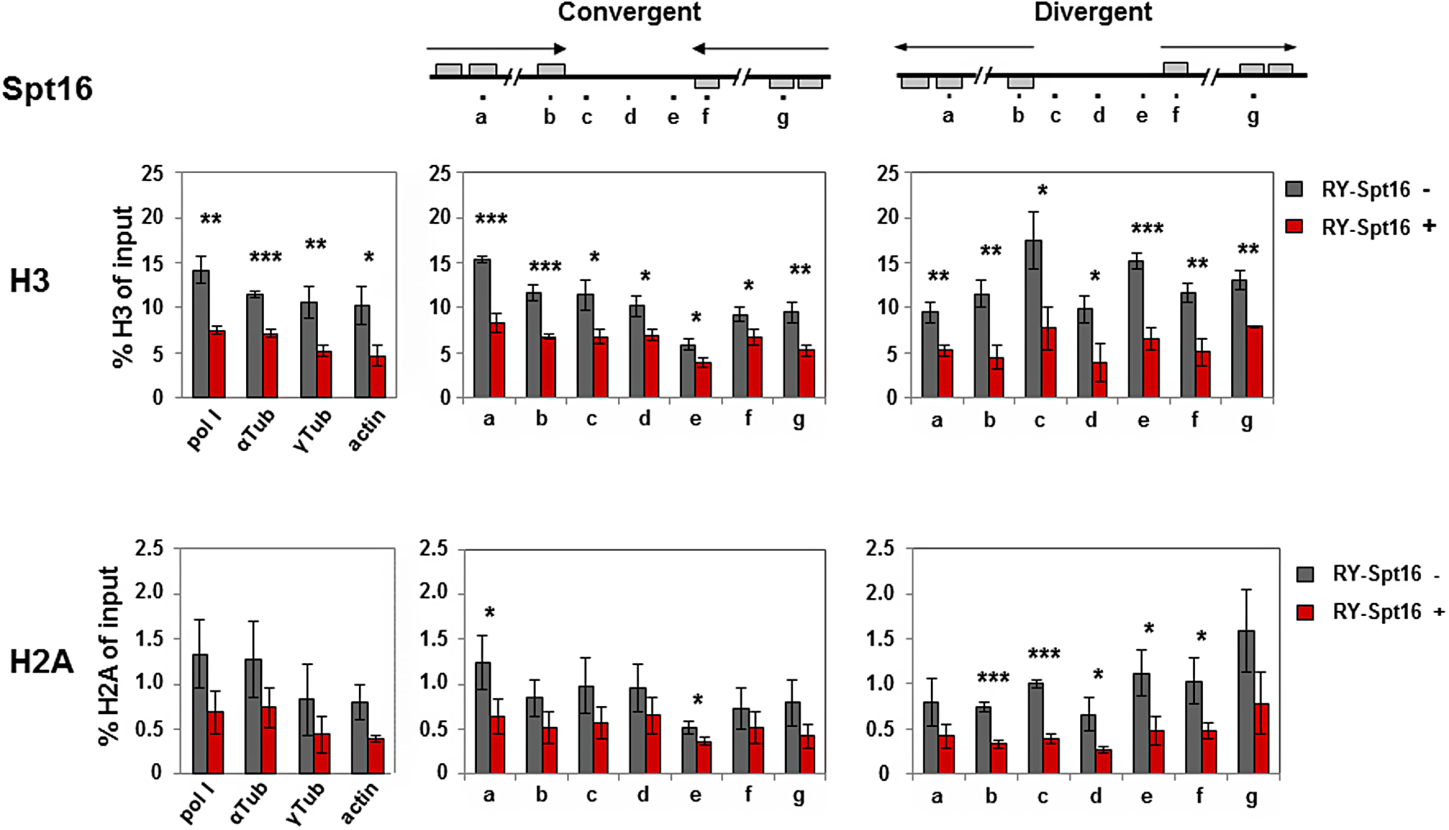
Knockdown of the FACT large subunit Spt16 results in decreased levels of histones H3 and H2A at Pol II transcription units and strand switch regions. The distribution of histones H3 and H2A was determined at Pol II transcription units and at strand switch regions (SSR), which were either convergent (containing putative Pol II terminators) or divergent (containing putative Pol II promoters) after Spt16 knockdown. Histone ChIP material was analysed by qPCR. The schematics show Pol II convergent and divergent SSRs (C2 and D1 of chromosome 10) with qPCR primers indicated with lettered dots as in Stanne *et al*. (2011). In the top bar graphs histone H3 levels were determined in the *T. brucei* RY-Spt16 strain in the presence (+) or absence (−) of tetracycline for 24 h to induce Spt16 RNAi. Pol II transcription units analysed include the large subunit of RNA polymerase I (pol I), α-tubulin (tub), γ-tubulin and actin, as well as the transcription units around the depicted SSRs. The mean of three independent experiments is shown, with the standard deviation indicated with error bars. Statistical significance was determined with significant: * (*P* = 0.01–0.05); very significant: ** (*P* = 0.001–0.01); extremely significant: *** (*P* < 0.001) indicated. The lower panel of bar graphs is as above, only distribution of histone H2A was determined.

In addition to playing a key role in facilitating transcription elongation of Pol I (Birch *et al*., 2009), FACT has also been reported to be crucial for Pol II transcription initiation and elongation, and for preventing transcription from cryptic sites (Kaplan *et al*., 2003; Mason and Struhl, 2003). However, unlike in other eukaryotes, Pol II transcription in *T. brucei* is not regulated (Clayton, 2002). This raises the question if histone chaperones and chromatin structure are functionally relevant for Pol II transcription in *T. brucei*. We therefore investigated levels of histone H3 and H2A after knockdown of the FACT subunit Spt16 at various Pol II transcribed loci as well as two strand switch regions (SSR) (Siegel *et al*., 2009; Stanne *et al*., 2011). Spt16 is evenly distributed over convergent and divergent SSRs (Fig. S5B). Depletion of Spt16 caused a very striking and significant loss of histones H3 and H2A within convergent and divergent SSRs, as well as within the Pol II transcription units themselves (Fig. 5, Tables S3 and S4). There was a minor but statistically significant decrease in tubulin precursor transcript after Spt16 knockdown, indicating possible downregulation of pol II transcription (Fig. S7, Table S5). Different histone variants have been shown to be enriched at *T. brucei* SSRs (Siegel *et al*., 2009), and it is not known if the observed effects on Pol II chromatin structure and transcription after FACT knockdown are a consequence of its activity on the specific variant nucleosomes found at these loci. This decrease in histones was not a general consequence of lethality, as in general knockdown of Spt6 did not have a significant effect on H3 and H2A levels at these Pol II loci for most of the regions that were analysed (Fig. S8).

### Cell cycle arrest at G2/M does not automatically result in derepression of ESs

Depletion of Spt16 in bloodstream-form *T. brucei* results in an unusually precise cell cycle arrest at G2/M, with an accumulation of cells which have replicated the nuclear genome, but not undergone mitosis (1N2K) (Denninger *et al*., 2010). This cell cycle arrest is also correlated with derepression of the silent *VSG221* ES. This raised the possibility that *VSG* ES derepression always transiently occurs at this precise cell cycle stage, rather than being a consequence of an additional specific chromatin remodelling event. Cyclin 6 has been reported to be crucial for the progression of mitosis in *T. brucei,* and its knockdown results in a G2/M cell cycle arrest as observed after knockdown of Spt16 (Hammarton *et al*., 2003; 2007[Bibr b29],[Bibr b30]; Hammarton, 2007) (Figs S9 and S10). Knockdown of Cyclin 6 leads to an immediate growth arrest (Fig. S9). This is accompanied by an increase in cells in the G2/M cell cycle phase, reaching a maximum of about 70% after 12 h induction of RNAi (Fig. S10). These results are in agreement with (Hammarton *et al*., 2003), and show a comparable degree of accumulation of cells in G2/M as observed after the induction of Spt16 RNAi for 24 h (Fig. S10) (Denninger *et al*., 2010).

**Figure 6:**
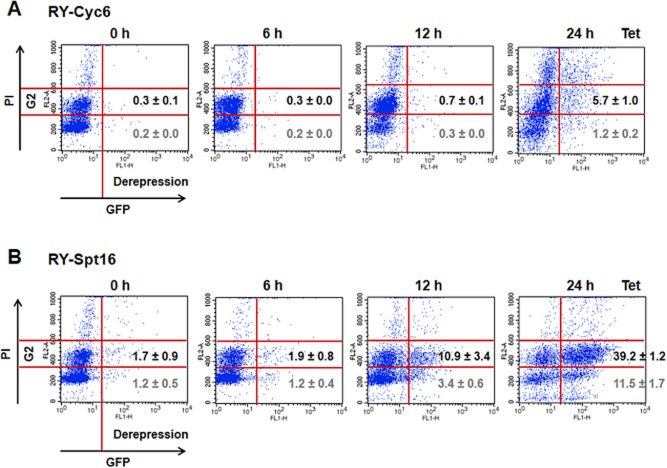
Depletion of cyclin 6 and Spt16 results in a comparable accumulation of cells at the G2/M cell cycle stage (4n DNA content), but only depletion of Spt16 leads to derepression of a silent *VSG* ES promoter.A. Knockdown of cyclin 6 does not result in significant derepression of silent ES promoters. Cyc6 RNAi was performed in the *T. brucei* RYT3 reporter cell line (Hughes *et al*., 2007), which has a GFP gene immediately downstream of the promoter of the silent *VSG221* ES. Cells were harvested after induction of Cyc6 RNAi with tetracycline (Tet) for the time indicated in hours (h). Cells were stained with propidium iodide (PI) and subjected to flow cytometry. Derepression of GFP behind the silent *VSG221* ES promoter was monitored in the FL1 channel (*x*-axis), with values over 20 considered significant ES promoter derepression. The DNA content was monitored in the FL2 channel (*y*-axis) with the G2 gate indicating cells with 4n DNA content. The degree of ES promoter derepression in cells stalled in G2/M is shown, with the values corresponding to the mean of three independent experiments with the standard deviation.B. Knockdown of Spt16 results in significant derepression of ES promoters specifically at the G2/M cell cycle stage. Further the experiments in this panel are as described above.

In order to investigate the role of cell cycle arrest in ES control, we monitored *VSG* ES derepression in cells stalled at G2/M either after blocking synthesis of cyclin 6 or Spt16 using flow cytometry. DNA content was monitored in the FL2 channel, and derepression of *GFP* located in a silent *VSG* ES was monitored in the FL1 channel. Depletion of both Cyclin 6 and Spt16 resulted in an increase in cells with 4n DNA content (Fig. 6). The difference in kinetics of the cell cycle arrest in the two knockdowns is presumably due to the different half- lives of the respective transcripts and proteins (Figs S9 and S10). Depletion of Cyclin 6 did not lead to significant ES derepression in the stalled G2/M cells (5.7% total). However as expected, depletion of Spt16 resulted in significant derepression of the *VSG221* ES in G2/M cells (39% total) (Fig. 6) (Denninger *et al*., 2010). These results indicate that the observed derepression of silent *VSG* ES promoters observed at G2/M after Spt16 knockdown is a specific consequence of FACT histone chaperone activity, rather than a non-specific consequence of general ES promoter derepression at the G2/M cell cycle stage.

### Depletion of Spt16 results in a decrease in core histones

**Figure 7:**
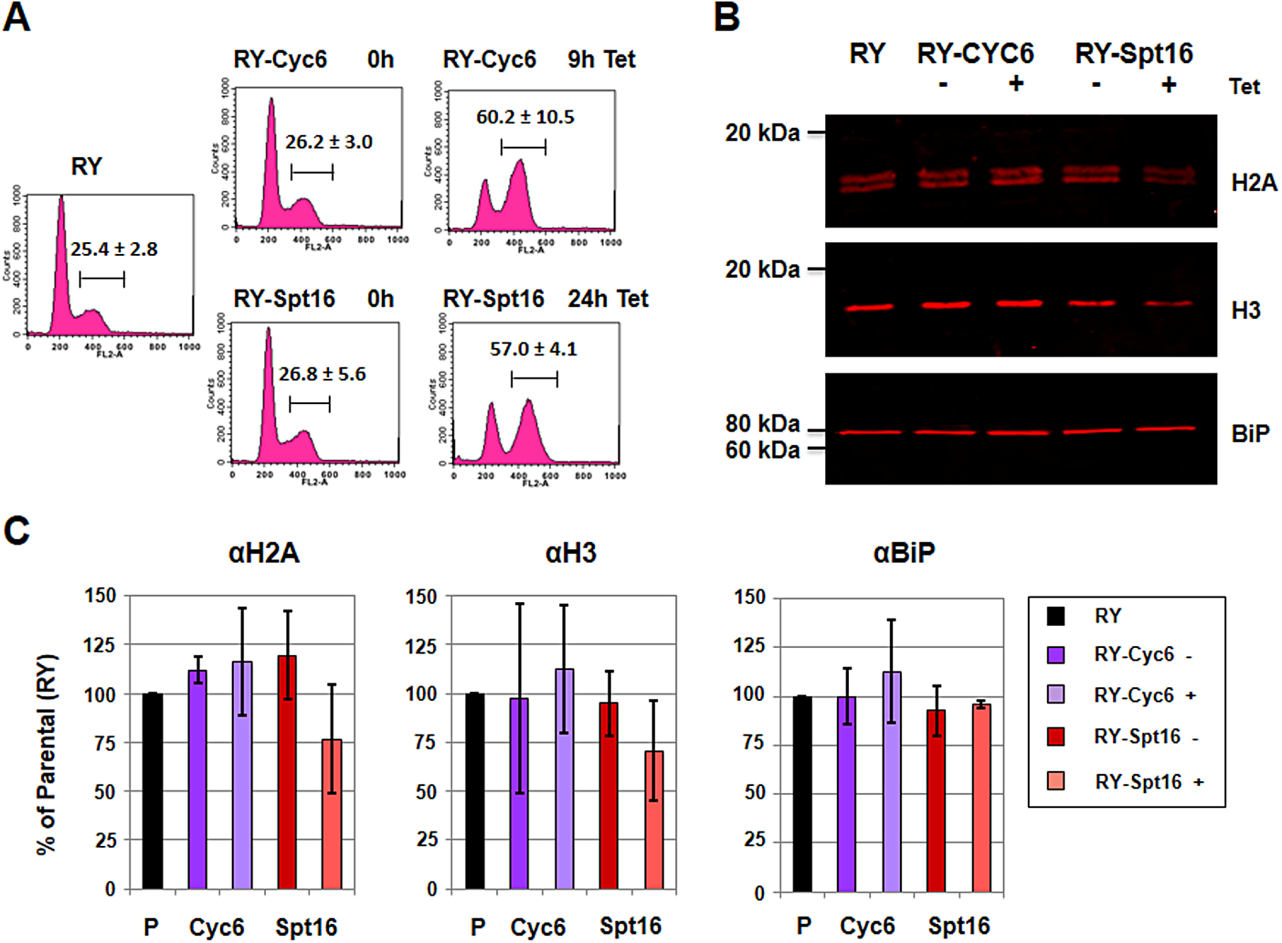
Depletion of the FACT large subunit Spt16 results in a decrease in the core histones H2A and H3.A. Cell cycle analysis after induction of cyclin 6 or Spt16 RNAi with tetracycline (Tet) for the time indicated in hours (h). The DNA content of cells stained with propidium iodide was measured by flow cytometry in the FL2 channel. The parental RY cell line is shown for comparison. After induction of cyclin 6 RNAi for 9 h, the percentage of cells with 4n DNA content is comparable to as observed after induction of Spt16-RNAi for 24 h. The gate containing cells in the G2/M phase of the cell cycle (4n DNA content) is indicated with a bar, with the mean value and standard deviation of three independent experiments shown.B. Quantification of histones H2A and H3 after depletion of Cyc6 or Spt16. Cells were grown in the presence (+) or absence (−) of Cyc6 or Spt16 RNAi induced with tetracycline (Tet). Representative Li-Cor Western blots of RY-Cyc6 cells after knockdown of cyclin 6 for 9 h or RY-Spt16 cells after knockdown of Spt16 for 24 h are compared with parental RY cells. Blots were probed for the core histones H2A and H3, and the loading control BiP before scanning with the Li-Cor system.C. Quantification of histone levels after induction of RNAi in the RY-Cyc6 or RY-Spt16 cells. The bar graphs show the relative level of histones after induction of Spt16 RNAi for 24 h or Cyc6 RNAi for 9 h compared with the parental RY strain. The histone signals from Western blots of three independent experiments were normalized to the parental strain, which was arbitrarily set at 100%.

Histone distribution is clearly disrupted after depletion of Spt16. With the exception of regions actively transcribed by pol I, ChIP of histones H3 and H2A revealed a significant decrease in histones bound to chromatin after Spt16 knockdown. We therefore monitored total levels of histones H3 and H2A (both soluble as well as chromatin bound) after depletion of Spt16. After the induction of either cyclin 6 or Spt16 RNAi, samples containing an approximately equivalent number of stalled cells were monitored for their cell cycle distribution using propidium iodide staining and flow cytometry (Fig. 7A). In order to precisely quantify overall histone levels, we used the Li-Cor Western blot imaging system, which provides a much broader linear range compared with standard chemiluminescence procedures. Blots were probed for histones H2A and H3 with BiP serving as a loading control (Fig. 7B). The signals with and without induction of RNAi were normalized to the parental RYT3 cell line which was arbitrarily set at 100%. Two bands were observed for histone H2A, presumably due to the presence of post-translational modifications. *T. brucei* histone H2A has been shown earlier to have various hyper-acetylated forms (Janzen *et al*., 2006).

After depletion of cyclin 6, levels of histones H2A and H3 increased as would be expected for a population with an increased percentage of cells with 4n DNA content (Fig. 7C, Table S6). However, interestingly, this was not the case after depletion of Spt16. Despite the fact that this too generated an increased proportion of cells with double DNA content, total levels of histone H3 and H2A appeared to decrease. There appeared to be a clear trend, although due to experimental variability these results were not statistically relevant. These results indicate that knockdown of the FACT histone chaperone Spt16 results in a disruption of chromatin structure correlated with a possible decrease in the overall level of core histones.

## Discussion

Here we gain insight into the degree of conservation in nucleosome dynamics in the ancient parasite *T. brucei* compared with other eukaryotes like yeast and mammals. We show that the *T. brucei* FACT complex is composed of the Pob3 and Spt16 subunits similar to in yeast, although a candidate for a DNA-binding protein like Nhp6 is missing. We demonstrate that the FACT complex plays a key role in ES silencing, whereby blocking its synthesis leads to an increase in transcription from silent ES promoters. This ES derepression is correlated with a reduction in the level of histones around silent ES promoters, and a relative opening of the chromatin structure. At the active ES, a block in FACT synthesis results in a relative increase in histones and a shut-down of transcription. The ES promoter derepression observed after FACT knockdown appears to be a direct consequence of perturbation of histone chaperone activity at the G2/M cell cycle stage, rather than an indirect effect resulting from general ES derepression at this cell cycle stage. The FACT complex in *T. brucei* therefore plays an important role in general chromatin dynamics in *T. brucei*, including histone distribution and nucleosome spacing.

In general in eukaryotes, FACT is thought to remove nucleosomes before the elongating RNA polymerase, and then reassemble them behind it after it has passed (Belotserkovskaya *et al*., 2003; Belotserkovskaya and Reinberg, 2004). This means that FACT knockdown can potentially generate different outcomes at different types of transcription units depending on the relative dynamics of these two processes (discussed in Formosa, 2012). Although FACT has an impact on both Pol I and Pol II transcribed areas in *T. brucei*, these different transcription units are affected in different ways. At inactive Pol I transcription units (silent VSG ES promoters), knockdown of Spt16 leads to localized depletion of histones. This is coupled with an increased sensitivity of these genomic regions to MNase digestion, and concurrent derepression of ES transcription (Denninger *et al*., 2010). This relative opening of chromatin structure at silent VSG ESs after FACT knockdown does not extend to the chromosome end, providing a possible explanation for why knockdown of Spt16 does not result in an increase in transcripts from the telomeric VSGs at the silent VSG ESs (Denninger *et al*., 2010).

In contrast, active Pol I transcription units including the active ES are already drastically depleted of histones (Figueiredo and Cross, 2010; Stanne and Rudenko, 2010). Here knockdown of Spt16 results in a relative increase in histone H3 on the active ES and a decrease in transcription, indicating that the observed increase in nucleosomes could be enough to disrupt Pol I (Denninger *et al*., 2010). FACT knockdown affects the Pol I transcribed rDNA loci in a fashion similar to as seen at the active ES. However, interpretation of phenotypes at these loci is complicated by the fact that presumably not all of the rDNA in *T. brucei* are transcriptionally active (McStay and Grummt, 2008). At Pol II transcription units in *T. brucei,* chromatin structure appears to be regulated differently to at Pol I transcription units. Pol II transcribed regions, do not show the extreme depletion of nucleosomes observed at active Pol I transcription units (Figueiredo and Cross, 2010; Stanne and Rudenko, 2010; Povelones *et al*., 2012). However, Spt16 knockdown does result in a reduction in histone abundance, and a decrease in levels of Pol II transcripts. This indicates that elongating Pol II is negatively affected by these changes in nucleosome abundance.

Among these different genomic loci, it is only at the silent ESs that knockdown of FACT resulted in a clear increase in sensitivity to MNase digestion. It has been shown earlier that silent ESs are present in relatively condensed chromatin, which requires histone H1 (Povelones *et al*., 2012; Pena *et al*., 2014). It is possible that this relatively compact heterochromatin also requires the FACT complex for maintenance. These variations in how these different transcription units react to knockdown of the FACT complex in *T. brucei* could be due to differences in how the different types of RNA polymerase react to changes in the dynamics of how nucleosomes are removed and reassembled around them.

It is unclear if these differing effects of FACT knockdown on different types of Pol I transcription units is specific to *T. brucei*. In other eukaryotes, the vast majority of analyses of the FACT complex concern its role in facilitating Pol II transcription (Formosa, 2012). In mammalian cells, the FACT complex has also been shown to facilitate transcription of RNA polymerase I and III, and its knockdown results in a reduction of rRNA precursor transcripts (Birch *et al*., 2009). As synthesis of the initial 40 nt of rRNA was not affected, the authors concluded that FACT is important for Pol I elongation through chromatin (Birch *et al*., 2009). However these authors did not investigate the impact of FACT knockdown on the silent rDNA transcription units.

One possible explanation for the differential role of FACT at different types of transcription units, is through specific interactions with different histone variants. Other studies have shown that FACT can interact with histone variants, such as H2AX and H2A.Z in mammalian cells and yeast (Heo *et al*., 2008; Mahapatra *et al*., 2011). TbH2AZ is one of the histone variants found in *T. brucei*, which dimerizes exclusively with TbH2BV, forming less stable nucleosomes. TbH2AZ/TbH2BV dimers were shown to be enriched at repetitive genomic regions including the telomeres, mini-chromosomal 177 bp repeats, 50 bp repeats and rDNA spacer, as well as the Pol II strand switch regions (Lowell *et al*., 2005; Siegel *et al*., 2009). If *T. brucei* FACT binds not only the core histones, but is also involved in remodelling the less stable nucleosomes formed by histone variants such as TbH2AZ and TbH2BV, this could possibly lead to its strong effect on the silencing of VSG ES promoters, nucleosome abundance at the Pol II strand switch regions as well as mini-chromosome segregation. This possible explanation for the role of FACT in *T. brucei* would require further investigation.

Although there is significant ES promoter derepression observed at the G2/M cell cycle stage after knockdown of FACT, this ES promoter derepression was not observed in cells arrested at G2/M after knockdown of cyclin 6. This argues that ES promoter derepression is specifically due to the histone chaperone activity of FACT at the G2/M cell cycle stage, rather than an indirect consequence triggered by the G2/M cell cycle checkpoint. ES promoter derepression therefore appears to be a direct consequence of disruption of chromatin structure (for example through local reduction in histone abundance) rather than linked to any specific cell cycle stage. Knockdown of the FACT large subunit Spt16 resulted in a highly significant decrease in histone H3 at silent ES promoters. It is likely that there is a causal relationship between this reduction in histone H3 at silent ES promoters at the G2/M cell cycle stage and ES promoter derepression. Evidence in support of this are experiments looking directly at the effect of knockdown of histone H3, where cells at G2/M show specific ES derepression (Alsford and Horn, 2012). This ES promoter derepression can therefore be mediated either through knockdown of the histone chaperone FACT as we show here, or directly through depletion of histone H3 itself.

Additional evidence that ES promoter derepression is not always linked with a specific cell cycle arrest, is that knockdown of some chromatin assembly factors can result in ES promoter derepression at different cell cycle stages. Depletion of the histone chaperone ASF1 did not result in a reduction in steady-state levels of histone H3, but ES promoter derepression was seen throughout the cell cycle (Alsford and Horn, 2012). There is also no evidence that knockdown of ISWI or NLP results in accumulation of cells with derepressed ES promoters at any particular cell cycle stage. Furthermore, knockdown of the histone chaperone CAF-1b resulted in ES derepression during both S phase and G2/M (Alsford and Horn, 2012).

In addition to the role of chromatin remodellers on ES control, disruption of DNA synthesis or chromosome segregation also results in ES promoter derepression or increased rates of VSG switching. Incubation of bloodstream-form *T. brucei* with the DNA synthesis inhibitor aphidicolin results in the accumulation of cells stalled at S phase, which show concurrent derepression of silent ES promoters (Sheader *et al*., 2004). In addition, it has been shown that knockdown of the DNA replication proteins ORC or MCM-BP leads to derepression of silent *VSG*s (Tiengwe *et al*., 2012; Benmerzouga *et al*., 2013; Kim *et al*., 2013). This argues for a connection between DNA replication and ES silencing. These cells stalled at S phase can be expected to have a large number of stalled replication forks, which might interfere with the establishment of a repressed chromatin state in a dividing trypanosome. Similarly, disruption of chromosome segregation through knockdown of the cohesin subunits resulted in increased VSG switching, although limited derepression of silent VSG expression site promoters was observed (Landeira *et al*., 2009; Denninger *et al*., 2010).

It should be mentioned that in all experimental approaches where ES promoter derepression was observed, transcription from derepressed ESs was only a fraction of that derived from an active ES. This could indicate that multiple layers of control suppress silent ESs. For example, knockdown of FACT results in derepression of silent ES promoters, but very limited switching to other ESs, indicating that the machinery mediating mono-allelic exclusion of ESs is still intact (Denninger *et al*., 2010). This is in contrast to studies where disruption of cohesion subunits leads to limited derepression of silent ES promoters, but increased ES switching (Landeira *et al*., 2009; Denninger *et al*., 2010).

FACT knockdown results in relative opening of the chromatin structure (decrease in levels of histone H3 and H2A and increased sensitivity to MNase) immediately around the silent ES promoter, but not down at the silent telomeric *VSG*. This argues that ES promoters and telomeres have distinct chromatin domains, which are affected in different ways by knockdown of histone chaperones and chromatin remodellers. In general, knockdown of chromatin remodellers (including ISWI and NLP) leads to significant transcriptional derepression of silent ES promoters, but relatively low levels of processive transcription extending down to the silent telomeric *VSG* (Hughes *et al*., 2007; Narayanan *et al*., 2011; Stanne *et al*., 2011). Possibly, silencing forces (repressive chromatin gradients) extend up from the telomere end, preventing fully processive transcription. This could argue that different mechanistic forces impact on silent ESs at either the ES promoter or ES telomere. It is still unknown which telomere-binding proteins could mediate this postulated repressive gradient at the chromosome ends. One promising telomere binding candidate RAP1 (Yang *et al*., 2009), is now thought to affect chromatin state predominantly in procyclic *T. brucei* (Pandya *et al*., 2013).

The ES promoter derepression that we observe after downregulation of FACT does not result in an increase in fully processive transcription extending down to the ES telomere end (Denninger *et al*., 2010). However, as FACT knockdown affects Pol I and Pol II transcription in general (Belotserkovskaya and Reinberg, 2004; Birch *et al*., 2009), this lack of transcriptional processivity is not necessarily ES related. Superficially, it appears to be a paradox that FACT knockdown can simultaneously result in derepression of silent ES promoters, as well as decreased transcription of the active ES (Denninger *et al*., 2010). However, in other eukaryotes, FACT plays different roles dependent on the context of chromatin state, stability of nucleosome positioning and transcriptional activation state. It has been reported that FACT in yeast works in a close relationship with Pol II within constitutively transcribed areas of the genome. FACT transiently facilitates passage of Pol II during transcription, followed by rapidly re-establishing nucleosomes after passage of the polymerase, and thereby helping to stabilize the chromatin structure of the transcription units (Belotserkovskaya *et al*., 2003).

This is in contrast to the role of FACT during initial activation of previously silent promoters like GAL1 or HO in *S. cerevisiae*, where the FACT complex promotes rapid destabilization of the nucleosomal interface to activate the gene (Takahata *et al*., 2009; reviewed in Formosa, 2012). As discussed by Formosa (2012), FACT plays a key role in establishing repressive chromatin states, where its knockdown can result in transcriptional derepression. However, FACT is also necessary to overcome these repressive chromatin states, meaning that knockdown results in a reduction in transcription activation. This apparent inconsistency can be understood if one takes into account the reversibility of the core nucleosome reorganizing activity of FACT (Formosa, 2012).

Histone chaperones are not only important for assembling and disassembling nucleosomes during transcription, replication or DNA repair, but also for preventing non-nucleosomal interactions between histones and DNA (Laskey and Earnshaw, 1980; Andrews *et al*., 2010; Burgess and Zhang, 2013). In this context histone chaperones can function as a ‘storage room’ for the positively charged histones. They prevent histones from binding randomly to the negatively charged DNA, which would be catastrophic for the cell. Even during DNA replication, only a negligible percentage of the core histones can be found in the soluble fraction, with the majority bound either to DNA or to histone chaperones (Bonner *et al*., 1988; Hondele and Ladurner, 2011). This explains why cells have developed means to immediately degrade excess soluble histones in a regulated manner before they can lead to irreparable damage (Gunjan and Verreault, 2003; Gunjan *et al*., 2006). We show here that in *T. brucei* depletion of the FACT subunit Spt16 leads to loss of nucleosomes at Pol I and Pol II transcription units. In agreement with this, total levels of the core histones H2A and H3 decrease after depletion of FACT. A similar observation was made in both mammalian cells and yeast after depletion of either Nhp6 or HMGB1, which affect nucleosome assembly (Celona *et al*., 2011). Although cells remained viable, levels of core and linker histones were reduced to about 65% normal levels after knockdown of Nhp6. The extent of this effect was not equally distributed over the whole genome, but differed according to genomic locus (Celona *et al*., 2011; Mejia, 2011).

FACT has been reported to interact with nucleosomes as well as with free histones, and preventing accumulation of the latter (Morillo-Huesca *et al*., 2010; Winkler *et al*., 2011; Formosa, 2012). In yeast, the increase in free histones due to dysfunctional FACT triggers a checkpoint involving the cyclin CLN3 resulting in cells stalling at the G1 cell cycle phase before DNA replication (Morillo-Huesca *et al*., 2010). Bloodstream-form *T. brucei* appears to lack this checkpoint and undergoes a full DNA replication cycle before finally stalling at the G2/M cell cycle phase (Denninger *et al*., 2010). This has a striking resemblance to the effect after block of H2B and H4 synthesis in yeast, where cells become arrested in mitosis and fail to segregate their chromosomes (Han *et al*., 1987; Kim *et al*., 1988).

In conclusion, we show here that the FACT complex plays a key role in ES control in *T. brucei*. FACT knockdown leads to perturbation of the repressed chromatin state present at silent ES promoters but not telomeres, leading to transcriptional derepression. This derepressed ES transcription is specific to the G2/M stage of the cell cycle, arguing that histone chaperone activity at this cell cycle stage is key for ES silencing. The challenge now facing us is in elucidating the additional levels of control which mediate silencing operating from the ES telomere end, as well as which facilitate mono-allelic exclusion of the active *VSG*.

## Experimental procedures

### Trypanosome culture and genetic modification

Procyclic-form *T. brucei* WT 427 was cultured in SDM-79, supplemented with 10% fetal calf serum (FCS), 5 mg ml^−1^ haemin and the appropriate drugs at 27°C. Bloodstream-form *T. brucei* 427 was cultured in HMI-9 (Hirumi and Hirumi, 1989) containing 15% fetal calf serum at 37°C/5% CO_2_ and the relevant drugs. The RNAi constructs used in this study were transfected into the bloodstream-form *T. brucei VSGT3* strain RY-T3 (also called T3-SM) based on the ‘single-marker’ cell line (Wirtz *et al*., 1999; Hughes *et al*., 2007). The relevant RNAi fragments were cloned between opposing T7 promoters in the p2T7_hygro_RNAi construct which integrates into *T. brucei* minichromosomes (Wickstead *et al*., 2002). The RNAi fragments used contain: position 246–1027 of *Pob3* (GeneDB Tb927.10.14390), position 1405–2104 of *Spt6* (GeneDB Tb927.2.5810), position 10–722 of *Cyclin 6* (GeneDB Tb11.01.8460) and position 1408–2185 of *Spt16* (GeneDB Tb927.3.5620) (Denninger *et al*., 2010).

### Tandem affinity purification

The C-terminus of Spt16 was tagged with a ProtC-TEV protease site-ProtA (PTP) – epitope using the pC-PTP-hygro vector (Schimanski *et al*., 2005). The Spt16 C-terminus (final 804 bp) was amplified using the primers Spt16_2234s_PTP 5′-CAGT**GGGCCC**CTGAAGTTTGCTCAGGCCGTTGAAAGGG-3′ and Spt16_3036as_PTP 5′-CAAT**GCGGCCGC**CCAAACCGGCGCGGTGGCGG-3′. The construct was inserted into the *Spt16* locus via double cross-over. The tubulin 3′UTR of the pC-PTP-hygro vector was substituted by a part of the Spt16 3′UTR, which was amplified using the primers Spt16_DN_1s_tag 5′-CAA**GCATGC**TTAGGGTAGAGATGAAGAGGGAAAT-3′ and Spt16_DN_724as_tag 5′-GGT**ACTAG**GGAAAGCGCATAACCACATC-3′. After linearization with ApaI and SpeI, the construct was transfected into procyclic *T. brucei* 427.

To determine if Spt16-PTP was functional, the second Spt16 allele was knocked out using the pSpt16KO_phleo construct. Upstream and downstream Spt16 targeting regions were amplified using the primers Spt16_KO_UP_318s 5′-GAA**TCTAGA** TCCGTCTTTGTGGTGTAGGTC-3′ and Spt16_KO_UP_1as 5′-CAT**GGATCC**GCGAGATGCGGAAAGGGTAA-3′ and Spt16_DN_1s 5′-GAC**AAGCTT**TTAGGGTAGAGATGAAGAGGGAAAT-3′ and Spt16_DN_724as 5′-GTA**CTCGAG**GGAAAGCGCATAACCACATC-3′. Restriction sites are indicated in bold font.

For the protein affinity purification experiments, two litres of logarithmic stage procyclic Spt16-PTP cells were lysed on ice using a Dounce homogenizer, aliquoted and shock frozen in liquid nitrogen. Tandem affinity purification was performed according to Gunzl and Schimanski (2009). In short, proteins were extracted in ice-cold extraction buffer for 20 min on ice and cell lysates were centrifuged twice at 20 000 *g* at 2°C in order to separate extracted proteins from cell debris. The final supernatant was incubated with previously equilibrated IgG Sepharose Fast Flow beads (GE Healthcare) in the presence of protease inhibitors for 3.5 h at 4°C. After washing off the unbound proteins, Protein A was cleaved off Spt16-PTP using TEV-protease (Invitrogen). The TEV-eluate was subsequently bound to an anti-Prot C matrix (Roche) in the presence of Ca^2+^. Following a final wash step, Spt16-P was eluted from the column using EGTA and subjected to SDS-PAGE. The gel was stained using Imperial Protein Stain (Thermo Scientific), the bands cut out and analysed via mass spectrometry.

### Protein analysis

In order to prepare protein lysates, an equal number of cells per sample were centrifuged at 1000 *g* and washed twice. The cell pellet was resuspended in hot SDS sample buffer to a final concentration of 10^5^ cells μl^−1^, boiled for 5 min and centrifuged briefly. In order to quantify histone levels precisely, cell lysates were subjected to the ODYSSEY CLx Western blot imaging system (Li-Cor). Pilot experiments were performed to ensure that the obtained signal was indeed linear with the number of cells analysed. Subsequently, lysates containing an equal number of cells per sample were loaded on a 15% gel, which was subjected to Western blot analysis using standard procedures. A polyclonal anti-histone H3 antibody (Abcam) and an anti-histone H2A antibody (Millipore) were used to probe for the core histones. An antibody against BiP served as a loading control, and was a gift of Jay Bangs. The secondary antibody was a donkey anti-rabbit IRDye680 (Li-Cor). Imaging and quantification were performed using the ODYSSEY CLx system (Li-Cor).

### Flow cytometry

Cells were spun down at 1000 *g*, washed once in PBS, fixed in 2% paraformaldehyde for 2 h at room temperature before being washed again. For derepression analysis, cells were resuspended in PBS and immediately subjected to flow cytometry using a FACS Calibur. If additional cell cycle analysis was also performed, cells were permeabilized in 0.1% Triton-X for 10 min at room temperature. Cells were washed again, and resuspended in PBS containing 10 μg ml^−1^ RNase A and 10 μg ml^−1^ propidium iodide. After incubation for 45 min at 37°C, cells were subjected to flow cytometry. Per sample 100 000 cells were counted.

### qPCR quantification of RNA transcripts and precursors

Transcript analysis was performed as described in Denninger *et al*. (2010) with the following modifications: After induction of RNAi, cells were centrifuged at 1000 *g* and washed twice in PSG. Total RNA was isolated using the RNeasy Kit (QIAGEN). After DNase treatment using the TURBO DNA-free kit (Ambion), 500 ng RNA of each sample served as template for reverse transcription using the Omniscript RT Kit (QIAGEN). qPCR analysis was performed using the Brilliant II SYBR Green Mix (Stratagene) and the 7500 Fast Real-Time PCR System (ABI). All primers used are listed in Table S7. Amplification conditions for each primer pair have been optimized individually. An RNA sample that had not been reverse transcribed served as a negative control.

### Chromatin immunoprecipitation

In experiments where chromatin immunoprecipitation (ChIP) of the core histones was performed, Spt16 or Spt6 RNAi was induced in the *T. brucei* RYT3BSR2.1 cell line with 1 μg ml^−1^ tetracycline for 24 h. Cells were subsequently fixed in 1% formaldehyde for 1 h at room temperature. ChIP was performed essentially as described previously (Lowell and Cross, 2004; Stanne and Rudenko, 2010). A polyclonal rabbit anti-H3 antibody (Abcam) and a polyclonal anti-H2A antibody (Millipore) were used for the immunoprecipitation. The anti-H2A antibody was raised against the peptide IRNDEELNKL of the human H2A which cross-reacts with the corresponding peptide sequence VRHDDDLGAL of the *T. brucei* H2A (Blast searches in TriTryp show 50% sequence identity and 69% conservation between TbH2A and HsH2A). This peptide sequence is also highly conserved within *T. brucei* H2AZ (IRGDEELNQI), suggesting additional cross-reactivity of the antibody with this *T. brucei* histone variant. However, Western blot analysis demonstrated negligible detection of *T. brucei* H2AZ in comparison with *T. brucei* H2A. Protein A Sepharose CL-4B (GE Healthcare) served as matrix for binding the antibody-antigen complex. Histone occupancy on various loci was analysed via qPCR using Brilliant II SYBR Green Mix (Stratagene) and the 7500 Fast Real-Time PCR System (ABI). All primers used are either described in Stanne *et al*. (2011) (Divergent region D1 and Convergent region C2) or listed in Table S7. Conditions for each primer pair had been optimized individually. Values for no-antibody samples were subtracted in order to correct for the background noise.

### Micrococcal nuclease assay

In order to analyse micrococcal nuclease (MNase) digestion sensitivity using agarose gels, 8 × 10^7^ cells were centrifuged and washed once in pre-warmed PSG buffer. Each sample was then separated into four aliquots of 2 × 10^7^ cells. Cells were subsequently permeabilized, washed and resuspended in MNase buffer as previously described (Stanne and Rudenko, 2010). Each aliquot of 2 × 10^7^ cells was then incubated with micrococcal nuclease (Worthington Biochemicals) using 0.125 or 0.5 U for 5 min at 37°C. The samples were immediately put on ice, and the reaction was stopped with 10 mM EDTA and 10 mM EGTA. Following centrifugation at 10 000 *g* for 10 min at 4°C, the supernatant was discarded and the chromatin was solubilized by resuspending the nuclear pellet in 200 μl RSB buffer (Stanne and Rudenko, 2010). After another centrifugation step, the samples were treated by incubation with 0.2 mg ml^−1^ Proteinase K in 0.5% SDS for 1 h at 56°C and subsequently with 0.2 mg ml^−1^ RNase A for 30 min at 37°C. The samples were then phenol/chloroform extracted and ethanol precipitated. DNA pellets were dissolved in 30 μl Tris-buffer (pH 8), and 500 ng of each sample was analysed on a 2% agarose gel.

In order to separate nucleosomal fractions on a sucrose density gradient, the experiment was initiated with 3 × 10^8^ cells from induced and uninduced cultures. The procedure was performed according to Stanne and Rudenko (2010). The sucrose gradient fractions were purified by phenol/chloroform extraction and ethanol precipitation. Fractions enriched in mononucleosomes were pooled, as well as the remaining fractions with higher-order nucleosomes, and subjected to qPCR analysis. All primers used are listed in Table S7. The mononucleosome fraction was plotted as the percentage of total, which is the sum of all fractions (100%).

### Bioinformatics and statistical analysis

Blast searches were performed using SGD (Saccharomyces Genome Database) and TriTrypDB (Version 6.0). Protein sequence analysis and domain predictions were performed using SMART (Version 7.0) and InterProScan 5 (version 5–44.0).

Where indicated, the data were analysed via a Student's *t*-test (unpaired, two-tailed) using GraphPad Prism 5 software. Significant: * (*P* = 0.01–0.05); very significant: ** (*P* = 0.001–0.01); extremely significant: *** (*P* < 0.001).
