# Supplementary material for: FACT plays a major role in histone dynamics affecting VSG expression site control in Trypanosoma brucei
Source: Mol Microbiol. 2014 Oct 22;94(4):945–62. doi: 10.1111/mmi.12812 (PMC4625058; doi:10.1111/mmi.12812)
Supplement: Supplementary file 1 — Supporting information [file mmi0094-0945-sd1.pdf]

Mass spectrometry of the FACT complex using Spt16 protein affinity purification

Protein	GeneID	Score
Spt16	Tb927.3.5620	10303
Hypothetical, conserved (Pob3)	Tb927.10.14390	5838
Beta Tubulin	Tb927.1.2330	861
Alpha Tubulin	Tb927.1.2340	164
Histone H2A, putative	Tb927.7.2820	147
Histone H2B, putative	Tb927.10.10460	717
Histone H3, putative	Tb927.1.2430	102
Histone H4, putative	Tb927.5.4170	438

Sup Fig 1

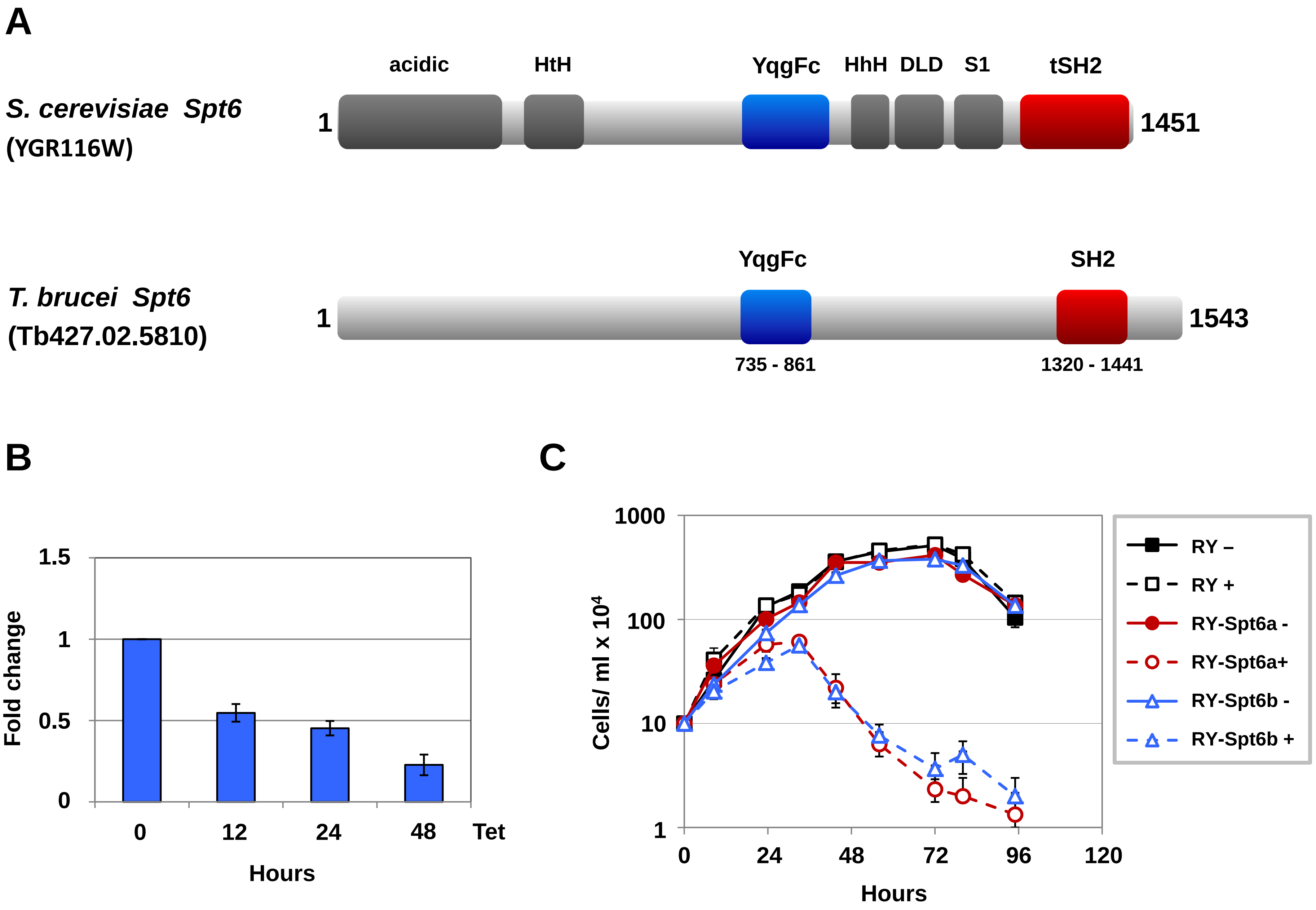
A
acidic
HtH
YqgFc
HhH
DLD
S1
tSH2
S. cerevisiae Spt6
(YGR116W)
1
1451
T. brucei Spt6
(Tb427.02.5810)
YqgFc
SH2
1
1543
735 - 861
1320 - 1441
B
Fold change
1.5
1
0.5
0
0
12
24
48
Tet
Hours
C
Cells/ ml x 10^4
1000
100
10
1
0
24
48
72
96
120
Hours
RY –
RY +
RY-Spt6a -
RY-Spt6a+
RY-Spt6b -
RY-Spt6b +

Sup Fig 2

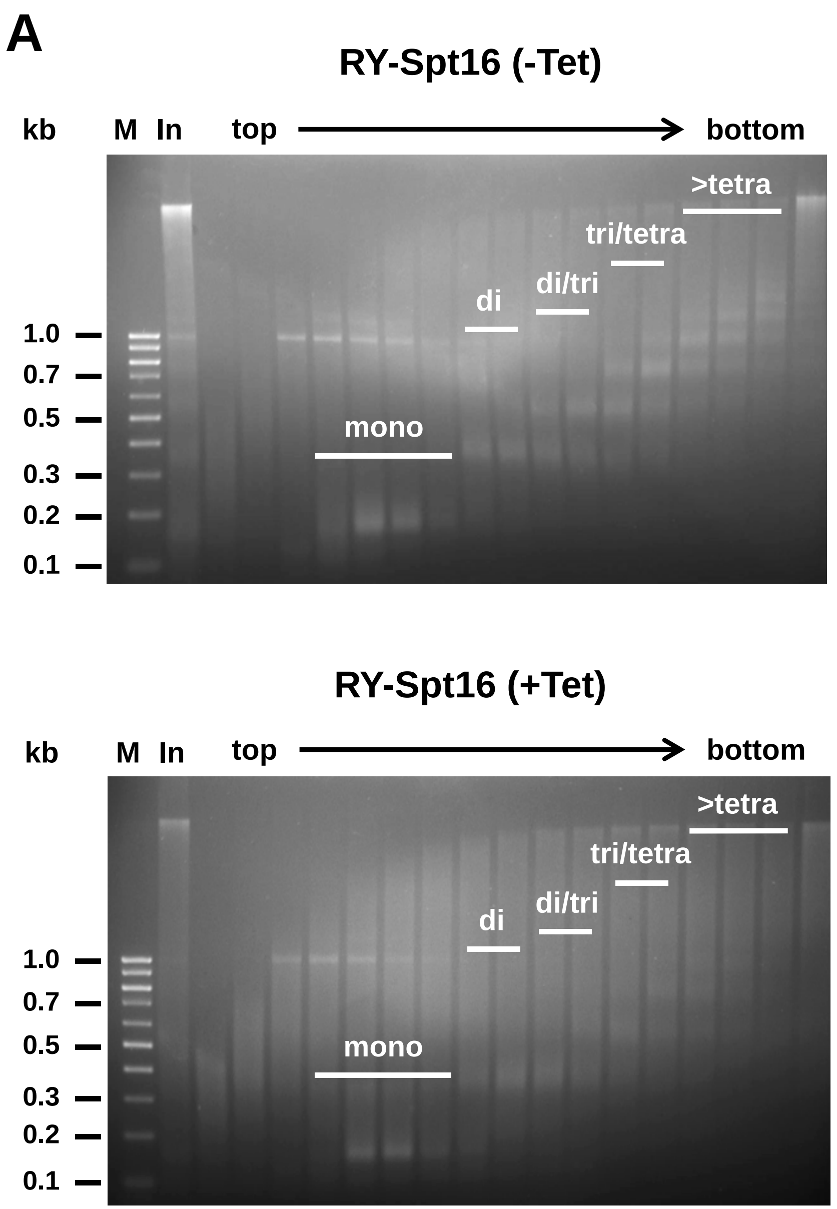
A
RY-Spt16 (-Tet)
kb
M
In
top
bottom
>tetra
tri/tetra
di/tri
di
mono
1.0
0.7
0.5
0.3
0.2
0.1
RY-Spt16 (+Tet)
kb
M
In
top
bottom
>tetra
tri/tetra
di/tri
di
mono
1.0
0.7
0.5
0.3
0.2
0.1

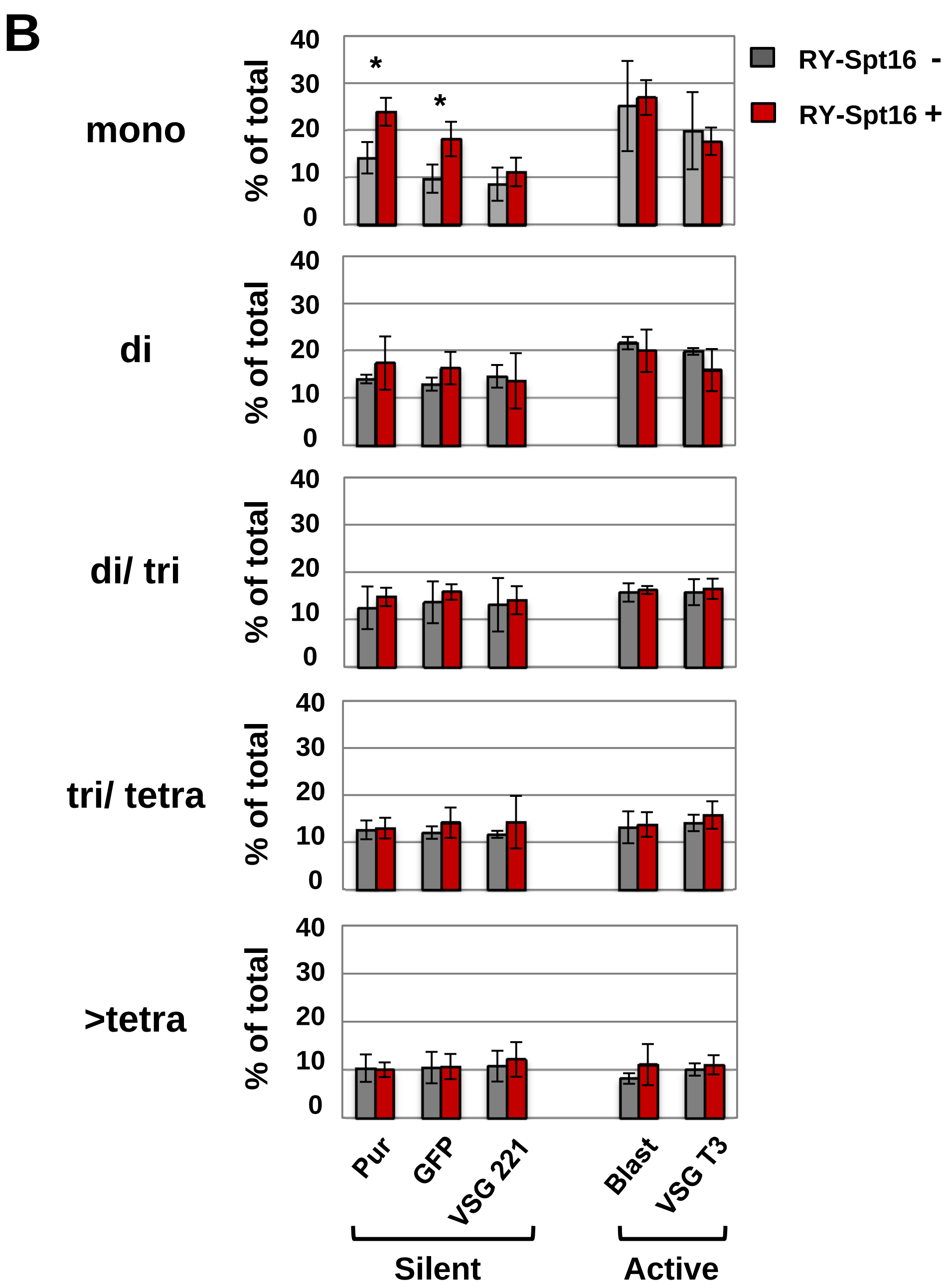
B
mono
di
di/ tri
tri/ tetra
>tetra
% of total
40
30
20
10
0
*
RY-Spt16 -
RY-Spt16 +
Pur
GFP
VSG 221
Blast
VSG T3
Silent
Active

Sup Fig 3

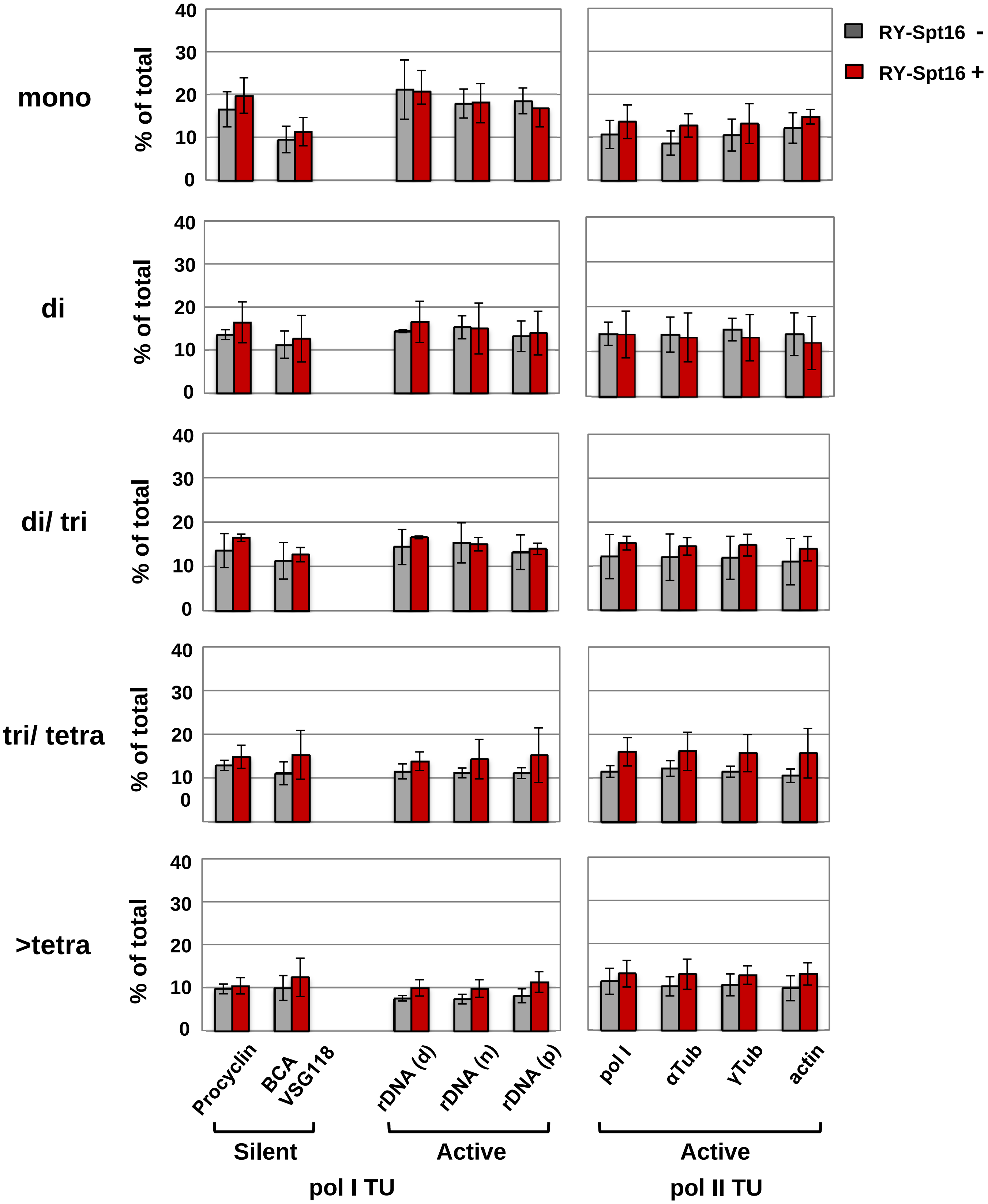

RY-Spt16 -
RY-Spt16 +
mono
di
di/ tri
tri/ tetra
>tetra
% of total
0
10
20
30
40
Procyclin
BCA
VSG118
rDNA (d)
rDNA (n)
rDNA (p)
pol I
αTub
γTub
actin
Silent
Active
pol I TU
Active
pol II TU

Sup Fig 4

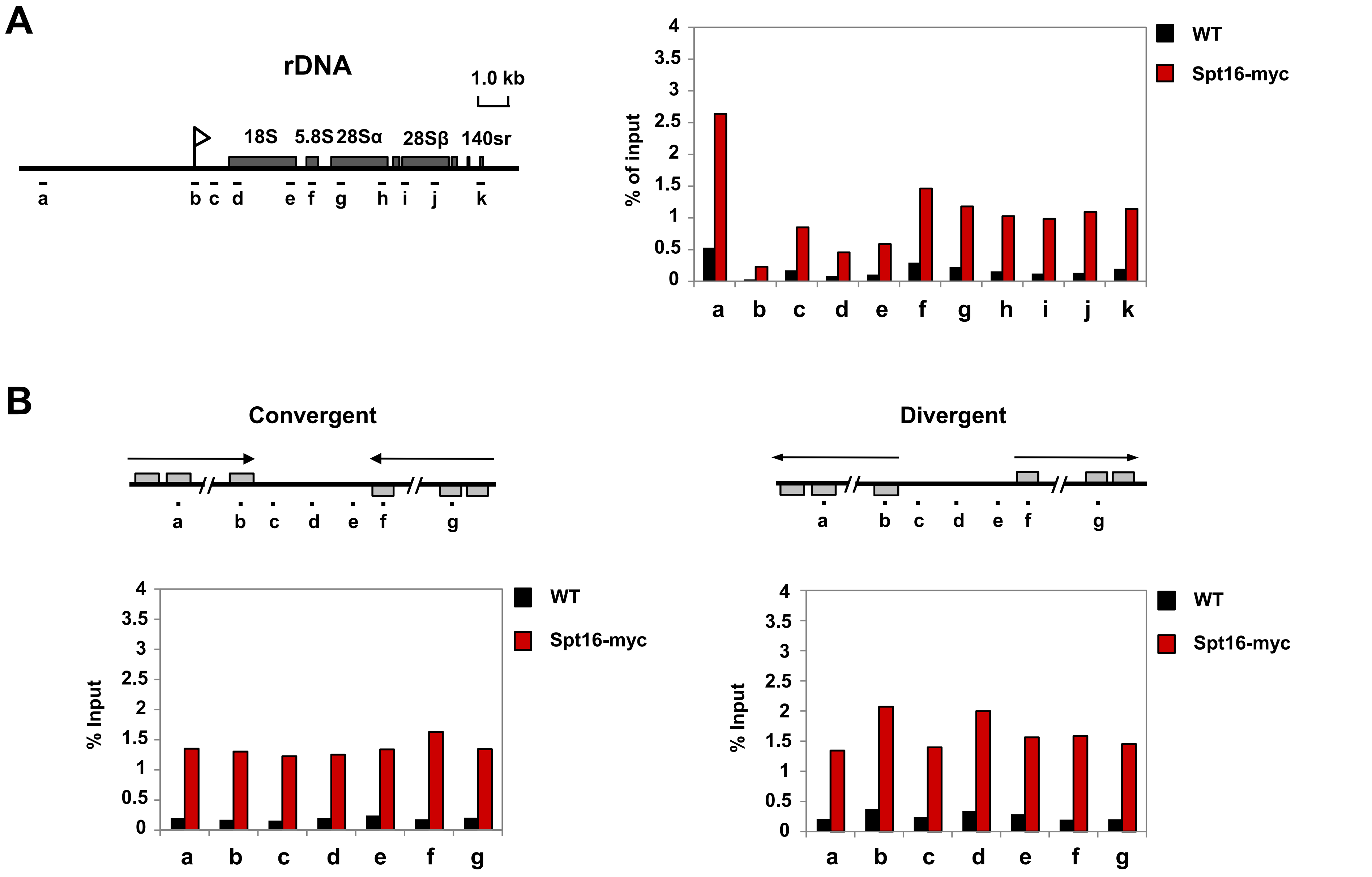
A
rDNA
1.0 kb
18S
5.8S
28Sα
28Sβ
140sr
a b c d e f g h i j k
% of input
4
3.5
3
2.5
2
1.5
1
0.5
0
WT
Spt16-myc
B
Convergent
Divergent
a b c d e f g
% Input

Sup Fig 5

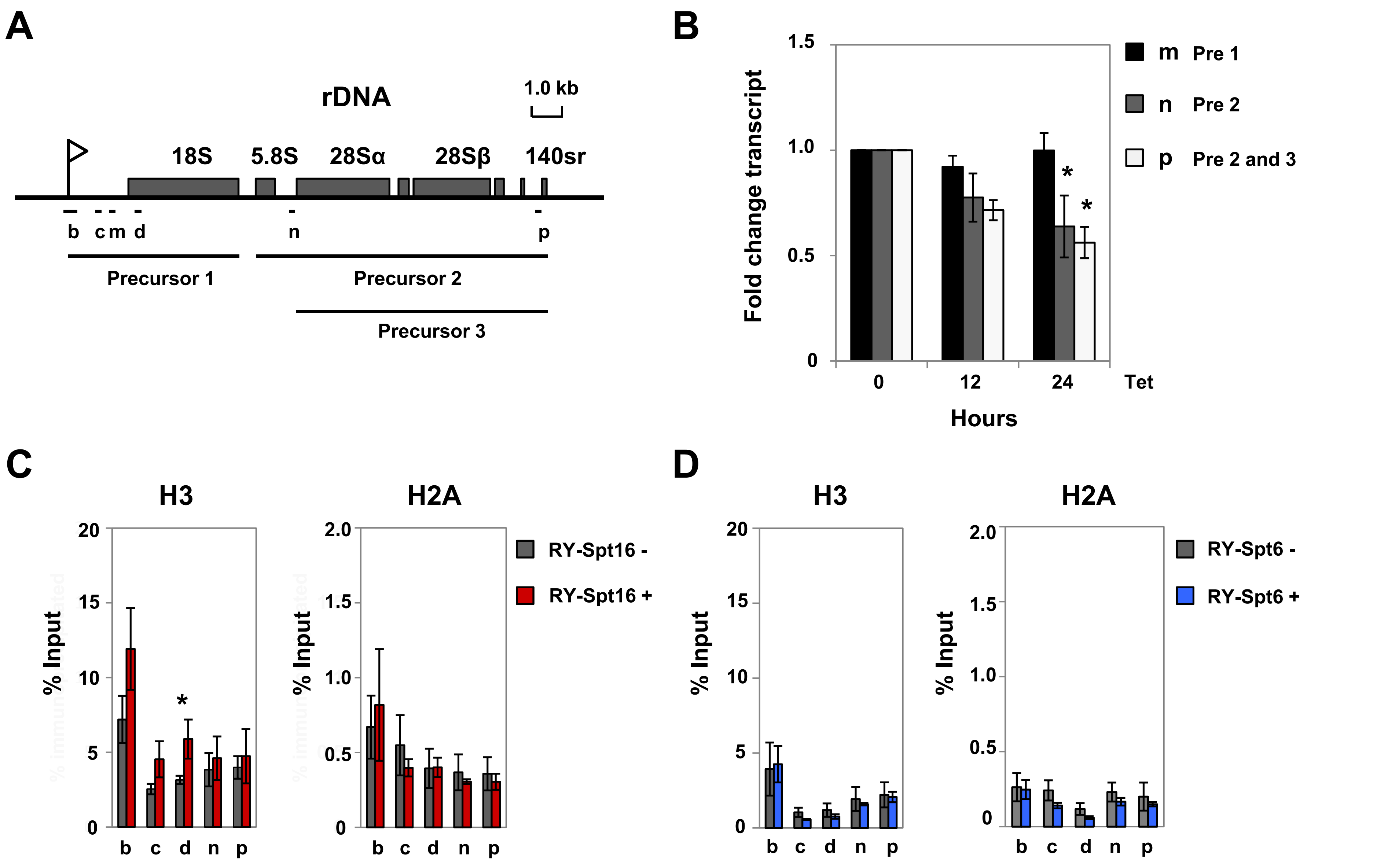
A
rDNA
1.0 kb
18S
5.8S
28Sα
28Sβ
140sr
b
c m
d
n
p
Precursor 1
Precursor 2
Precursor 3
B
Fold change transcript
1.5
1.0
0.5
0
0
12
24
Tet
Hours
m Pre 1
n Pre 2
p Pre 2 and 3
C
H3
H2A
% Input
20
15
10
5
0
2.0
1.5
1.0
0.5
b c d n p
RY-Spt16 -
RY-Spt16 +
D
H3
H2A
RY-Spt6 -
RY-Spt6 +

Sup Fig 6

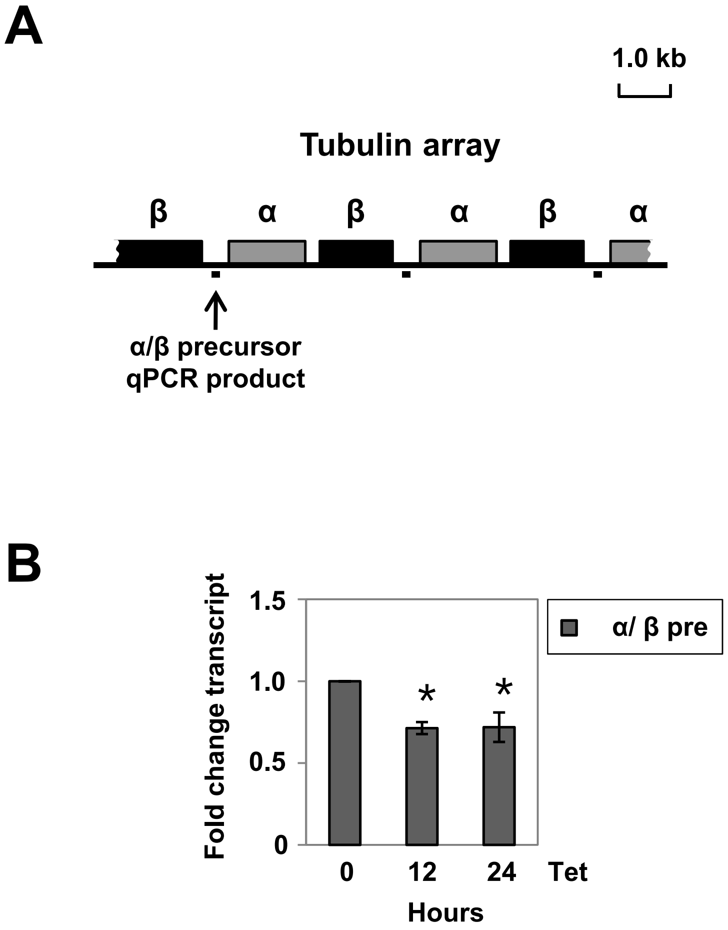
A
1.0 kb
Tubulin array
β
α
β
α
β
α
α/β precursor
qPCR product
B
Fold change transcript
1.5
1.0
0.5
0
*
*
α/ β pre
0
12
24
Tet
Hours

Sup Fig 7

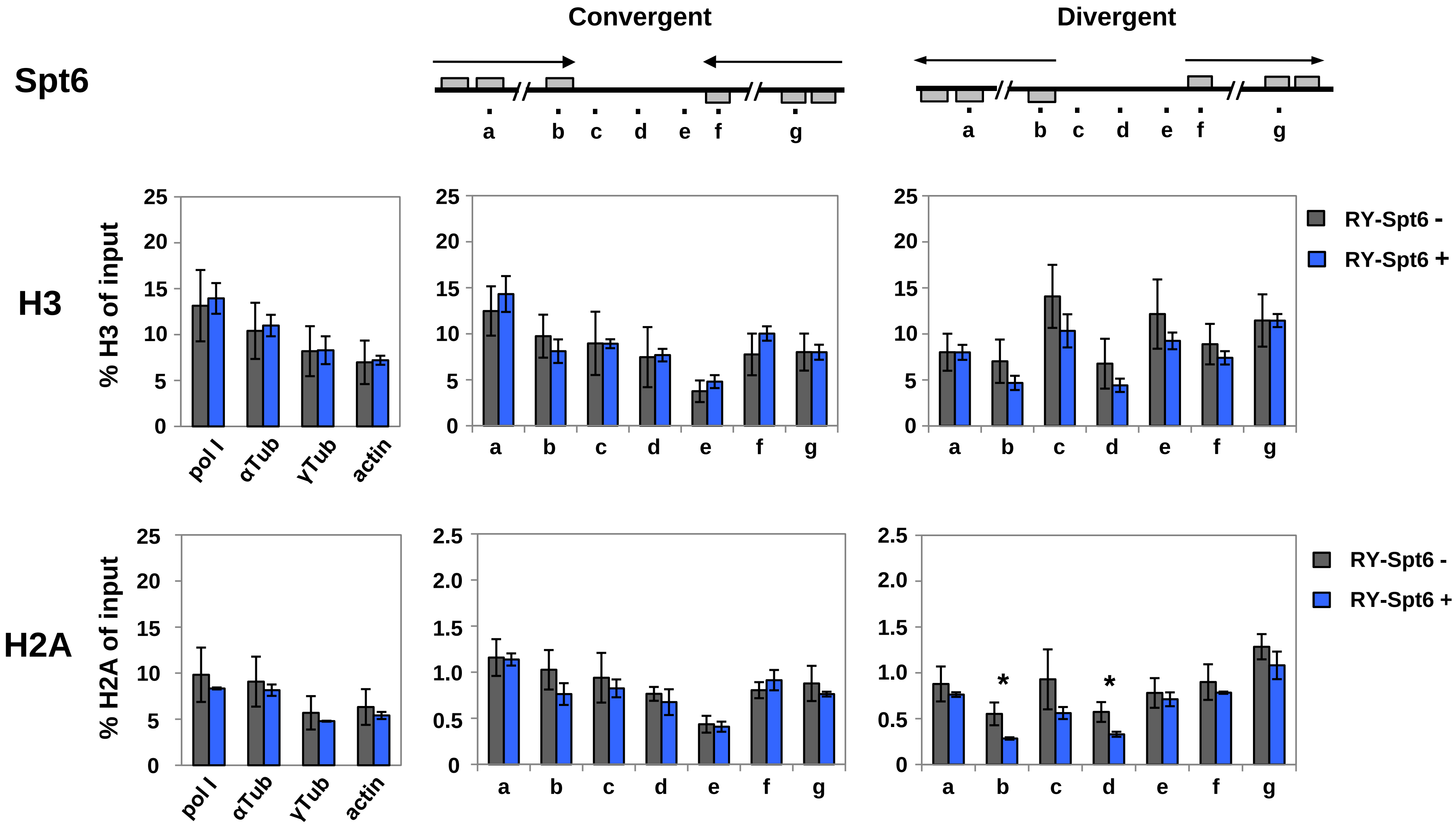

Convergent
Divergent
Spt6
a b c d e f g
a b c d e f g
H3
% H3 of input
25 20 15 10 5 0
pol I αTub γTub actin
RY-Spt6 -
RY-Spt6 +
H2A
% H2A of input
2.5 2.0 1.5 1.0 0.5 0
*
*

Sup Fig 8

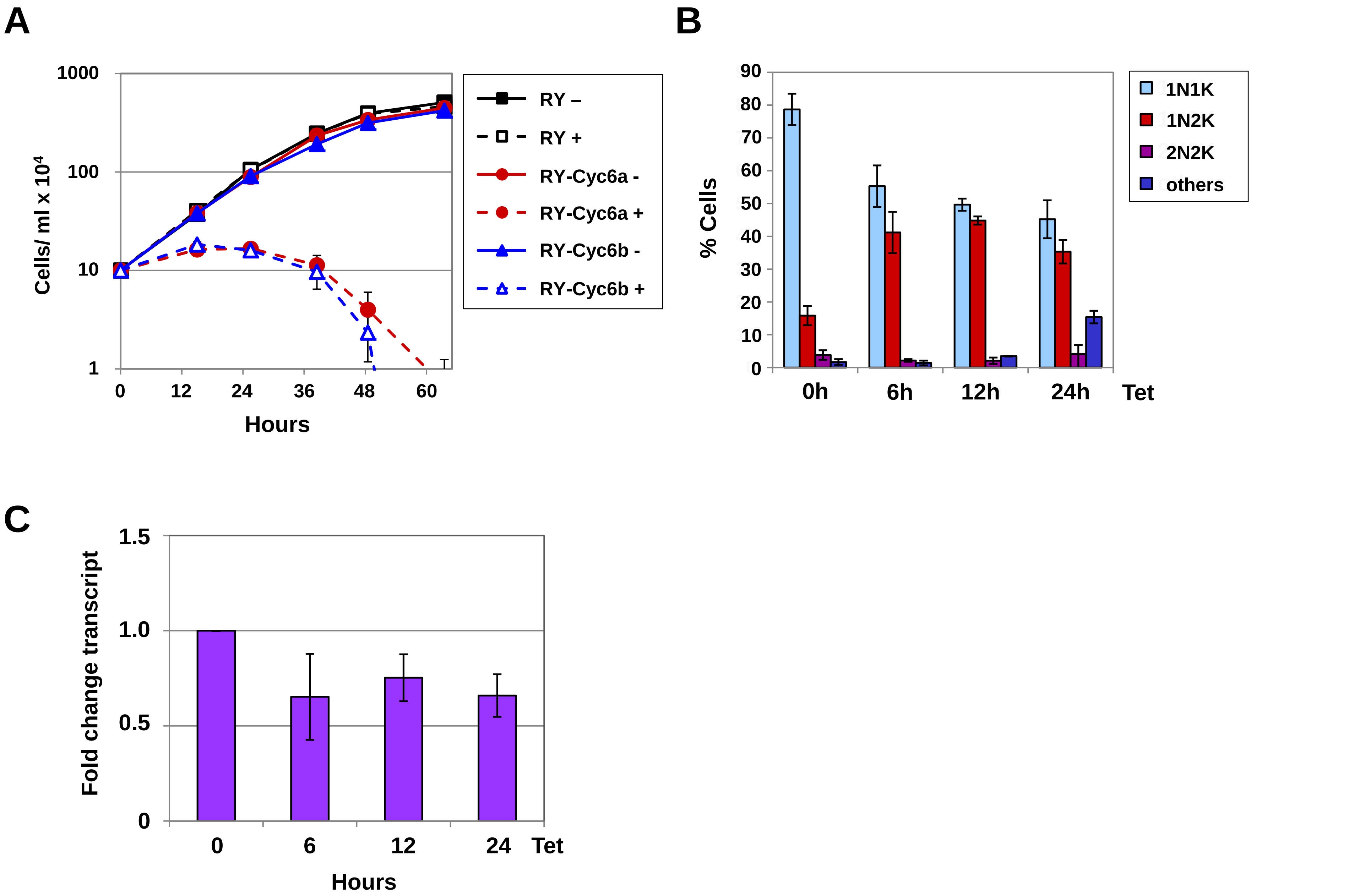
A
1000
100
10
1
Cells/ ml x 10^4
0
12
24
36
48
60
Hours
RY –
RY +
RY-Cyc6a -
RY-Cyc6a +
RY-Cyc6b -
RY-Cyc6b +
B
90
80
70
60
50
40
30
20
10
0
% Cells
0h
6h
12h
24h
Tet
1N1K
1N2K
2N2K
others
C
1.5
1.0
0.5
0
Fold change transcript
0
6
12
24
Tet
Hours

Sup Fig 9

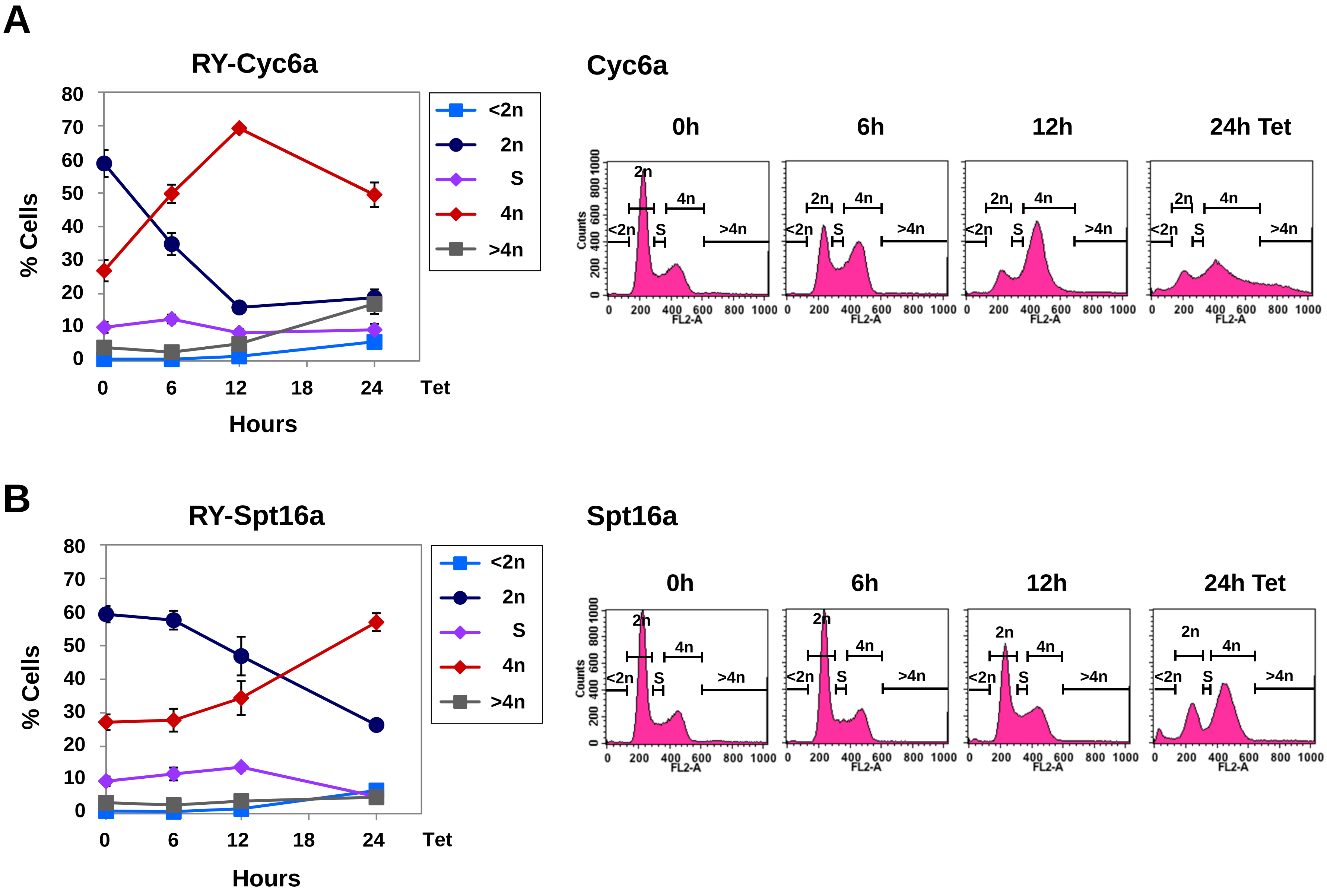
A
RY-Cyc6a
% Cells
80
70
60
50
40
30
20
10
0
0
6
12
18
24
Tet
Hours
<2n
2n
S
4n
>4n
Cyc6a
0h
6h
12h
24h Tet
Counts
FL2-A
B
RY-Spt16a
% Cells
Hours
Spt16a
0h
6h
12h
24h Tet

Sup Fig 10

Statistical significance:

Change in presence in mononucleosomal fraction after 24h vs 0h induction Spt16 RNAi:

Probe	P value	Significant
**Pur**	0.0193	Yes
**GFP**	0.0370	Yes
**VSG 221**	0.3915	No
**Blast**	0.7728	No
**VSG T3**	0.6794	No
**Procyclin**	0.3942	No
**BCA (VSG118)**	0.5205	No
**rDNA (d)**	0.9364	No
**rDNA (n)**	0.9246	No
**rDNA (p)**	0.6010	No
**Pol I**	0.3720	No
**α Tub**	0.1450	No
**γ Tub**	0.4773	No
**Actin**	0.3122	No

Sup. Table 1

Statistical analysis (t-test): Change in presence in nucleosomal fraction after 24 h vs 0 h induction Spt16 RNAi

Probe	Mono		Di		Di/Tri		Tri/Tetra		>Tetra		undigested	
	P value	Significant	P value	Significant	P value	Significant	P value	Significant	P value	Significant	P value	Significant
**Pur**	0.0193	Yes	0.3620	No	0.4607	No	0.8360	No	0.8642	No	0.1893	No
**GFP**	0.0370	Yes	0.1854	No	0.4659	No	0.3565	No	0.9318	No	0.2122	No
**VSG 221**	0.3921	No	0.8020	No	0.8054	No	0.4709	No	0.6420	No	0.6762	No
**Blast**	0.7726	No	0.5819	No	0.7074	No	0.8171	No	0.3228	No	0.0150	Yes
**VSG T3**	0.6784	No	0.2036	No	0.7448	No	0.4392	No	0.5183	No	0.7603	No
**Procyclin**	0.3937	No	0.6823	No	0.2742	No	0.3041	No	0.5974	No	0.4429	No
**BCA (VSG118)**	0.5191	No	0.7685	No	0.6120	No	0.3015	No	0.4647	No	0.5767	No
**rDNA (d)**	0.9363	No	0.1076	No	0.3946	No	0.2171	No	0.1028	No	0.9260	No
**rDNA (n)**	0.9253	No	0.2408	No	0.9211	No	0.3057	No	0.1417	No	0.9677	No
**rDNA (p)**	0.6014	No	0.3145	No	0.7682	No	0.3296	No	0.1302	No	0.8694	No
**Pol I**	0.3714	No	0.9665	No	0.3704	No	0.0914	No	0.5210	No	0.3818	No
**α Tub**	0.1452	No	0.8784	No	0.4859	No	0.2234	No	0.3066	No	0.3933	No
**γ Tub**	0.4766	No	0.6136	No	0.4121	No	0.1729	No	0.3037	No	0.4712	No
**Actin**	0.3122	No	0.6796	No	0.4398	No	0.2035	No	0.2094	No	0.4347	No

Sup. Table 2

Statistical significance
Change in histone H3 ChIP after induction RNAi for 24 h vs 0 h

	RY - Spt16		RY - Spt6	
**Probe**	**P value**	**Significant**	**P value**	**Significant**
**Pur**	0.0134	Yes	0.5041	No
**GFP**	0.0006	Yes	0.7734	No
**VSG 221**	0.1283	No	0.9144	No
**Blast**	0.1349	No	0.4322	No
**VSG T3**	0.0076	Yes	0.3555	No
**Pol I**	0.0017	Yes	0.7626	No
**α Tub**	0.0001	Yes	0.7733	No
**γ Tub**	0.0069	Yes	0.9554	No
**Actin**	0.0160	Yes	0.8807	No
**C a**	0.0003	Yes	0.3886	No
**C b**	0.0009	Yes	0.3491	No
**C c**	0.0133	Yes	0.9858	No
**C d**	0.0172	Yes	0.9125	No
**C e**	0.0165	Yes	0.2543	No
**C f**	0.0224	Yes	0.1764	No
**C g**	0.0049	Yes	0.9891	No
**D a**	0.0049	Yes	0.9891	No
**D b**	0.0036	Yes	0.1753	No
**D c**	0.0128	Yes	0.1694	No
**D d**	0.0159	Yes	0.2199	No
**D e**	0.0005	Yes	0.2621	No
**D f**	0.0011	Yes	0.3290	No
**D g**	0.0032	Yes	0.9969	No
**rDNA (b)**	0.609	No	0.8085	No
**rDNA (c)**	0.0520	No	0.0596	No
**rDNA (d)**	0.0240	Yes	0.1872	No
**rDNA (n)**	0.5103	No	0.4949	No
**rDNA (p)**	0.5454	No	0.7983	No

Sup. Table 3

Statistical significance:
Change in histone H2A ChIP after induction RNAi for 24 h vs 0 h

	RY - Spt16		RY - Spt6	
**Probe**	**P value**	**Significant**	**P value**	**Significant**
**Pur**	0.0359	Yes	0.0625	No
**GFP**	0.0363	Yes	0.0993	No
**VSG 221**	0.5192	No	0.1835	No
**Blast**	0.2904	No	0.6516	No
**VSG T3**	0.9590	No	0.0105	Yes
**Pol I**	0.0636	No	0.4313	No
**α Tub**	0.1289	No	0.5953	No
**γ Tub**	0.2007	No	0.4374	No
**Actin**	0.0235	Yes	0.4660	No
**C a**	0.0441	Yes	0.8683	No
**C b**	0.1031	No	0.1351	No
**C c**	0.1195	No	0.5253	No
**C d**	0.1809	No	0.3817	No
**C e**	0.0315	Yes	0.6819	No
**C f**	0.2576	No	0.2521	No
**C g**	0.0982	No	0.3575	No
**D a**	0.1381	No	0.3575	No
**D b**	0.0004	Yes	0.0203	Yes
**D c**	0.0001	Yes	0.1295	No
**D d**	0.0219	Yes	0.0192	Yes
**D e**	0.0193	Yes	0.5365	No
**D f**	0.0225	Yes	0.3611	No
**D g**	0.0745	No	0.1604	No
**rDNA (b)**	0.5816	No	0.8200	No
**rDNA (c)**	0.2819	No	0.0661	No
**rDNA (d)**	0.9480	No	0.0722	No
**rDNA (n)**	0.4252	No	0.1801	No
**rDNA (p)**	0.4974	No	0.4052	No

Sup. Table 4

Statistical significance:

Change in levels rRNA precursor transcripts after induction Spt16 RNAi

Probe	(h) RNAi	P value	Significant
**rDNA pc1**	12 vs 0	0.3380	No
	24 vs 0	0.9542	No
**rDNA pc2**	12 vs 0	0.0699	No
	24 vs 0	0.0248	Yes
**rDNA pc2 and 3**	12 vs 0	0.0546	No
	24 vs 0	0.0145	Yes
**α/β Tubulin pc**	12 vs 0	0.0153	Yes
	24 vs 0	0.0389	Yes

Sup. Table 5

Statistical significance:

Li-Cor Western analysis of change in histone levels:

Probe	P value: RY vs			
	CYC6 – no Tet	CYC6 – 9h Tet	Spt16 – no Tet	Spt16 – 24h Tet
H1	0.1977	0.0715	0.4705	0.1615
H2A	0.8305	0.8795	0.7830	0.6340
H3	0.6041	0.7442	0.9620	0.3732
BiP	0.9980	0.7675	0.8826	0.9274

Sup. Table 6

Table of primer pairs used for qPCR:

Fig.	Primer Pair	Forward Primer	Sequence (5' → 3')	Reverse Primer	Sequence (5' → 3')
**1**	Pob3	Pob3_1243s	attgaagttgatgagtcggaac	Pob3_1343as	ccgtgataggaagtgaagcaa
**2-4**	Pur	Puromycin_288s	cgagttgagcggttcc	Puromycin_333as	gccttccatctgttgct
	GFP	eGFP_184s	gtgaccaccctgacctac	eGFP_324as	cttgtagttgccgtcgtc
	VSG221	VSG221_46s	ctagcccaagttcttccaattc	VSG221_207as	cctcattttctggattttgctc
	Blast	Blasticidin_213s	caacctgacttgtatcgtc	Blasticidin_337a	catcactgtccttcactatg
	VSG T3	VSGT3_675s	aaacgacgcaacctctatc	VSGT3_904as	ctatgtcttctgggcttga
	Procyclin	Procyclin_8s	ctcgttccctttatctactc	Procyclin_102as	cttgtcttctggtccttc
	VSG118	VSGBCA118_108s	ctgcaagatgtctaaggaac	VSGBCA118_193as	caagttctaggatctccgtta
	rDNA d	18S_117s	gcattactggataacttgg	18S_250as	gttctaatttcattcattcg
	rDNA n	28Sa_up_136s	aaagaggcggcggatagtg	28Sa_up_52as	acgaaagaagcacaagcacata
	rDNA p	140srRNA_up_89s	ttgtgtttctatgtgtgtgtgtaag	140srRNA_1as	cgtttggagagggacaaaatat
	Pol I	PolI 349 s	acttatatgccagacgaagtgg	PolI 486 as	tgtgaatctctgggaagttgtc
	αTub	alphaTub 56s	cctgctgggaattgttctg	alphaTub 169as	cagtctcagagaagaaggtgttg
	γTub	GammaTub_491s	aatcaccgtatttggacacaag	GammaTub_583s	gtttgagtgttcgctgaaagag
	Actin	Actin_1031s	gttccatcctctcatcacta	Actin_1091as	tcgtattcactcttcgttatc
**S2**	Spt6	Spt6_qPCR_417s	gatacaccggaatgatgctcta	Spt6_qPCR_536as	gtttcttcacggagacctacac
**S4, S5**	a	rDNAspacer_178s	attttctctacccctctctt	rDNAspacer_323as	atcatcgtatcattttcatc
	b	rDNAProm_333s	gtcaatacaacacacacaatagg	rDNAProm_610as	cttaactgaggaagtgtcata
	c	rDNAProm_938s	ataaaagggagttatagcgt	rDNAProm_1048as	gtacaacacaatccgttaag
	d	18S_117s	gcattactggataacttgg	18S_250as	gttctaatttcattcattcg
	e	18S_1965s	gaatcacgtagacccactt	18S_2060as	gtaatcggcacagtttgat
	f	5.8S_36s	ggatgacttggcttcctat	5.8S_95as	attgataccacttatcgcact
	g	28Salpha_226s	acacatttacaacccttcat	28Salpha_410as	ctatcggtcttcctactctat
	h	28Salpha_1737s	ttgttaggaaagtgaaggt	28Salpha_1840as	aatatcatcccaggttctc
	i	28Sbeta_103s	gtaagttcgcaagaagcat	28Sbeta_292as	accagaaggaggttagtagata
	j	28Sbeta_1143s	gtaaatgcttgcttgatta	28Sbeta_1253as	tgacacctctggtatgatt
	k	rDNASp_27s	tatgtgtatgtgtgttgtgtta	rDNASp_108as	atgcaaaataggagactaca
	m	18S_up_377s	ccatgctctctcgtgtgtgta	18S_up_265as	ttcctcaaggcgtcactctatc
	n	28Sa_up_136s	aaagaggcggcggatagtg	28Sa_up_52as	acgaaagaagcacaagcacata
	p	140srRNA_up_89s	ttgtgtttctatgtgtgtgtgtaag	140srRNA_1as	cgtttggagagggacaaaatat
**S7**	α/β prec.	aTub_up_331s	gaggaggtgggaagggtatatg	aTub_up_205as	gaaggcgtgttgatgagttgta
**S8**	CYC6	Cyc6_699s	gccgtttcctttcattgttc	Cyc6_811as	gcatttgtgtctgttgagattg

Sup. Table 7

Figure legends supplementary figures

Sup. Fig. 1

Proteins found to interact with the FACT Spt16 large subunit using tandem affinity purification and subsequent mass spectrometry. A list of proteins with significant matches arranged according to their highest score as determined by mass spectrometry of bands isolated from SDS-PAGE gels. Only the FACT small subunit Pob3 was considered a significant interaction partner.

Sup. Fig. 2

The putative histone chaperone Spt6 is essential in bloodstream form *T. brucei*.

A. Comparison of *S. cerevisiae* Spt6 with the *T. brucei* orthologue, which was found by BLAST using the TriTrypDB, version 6.0 database. For *T. brucei* 427 the e-value is $4.1e^{-07}$ and for *T. brucei* 927 the e-value is: $3.2e^{-07}$. Of the multiple domains present in Spt6 in *S. cerevisiae* and higher eukaryotes, only the YqgFc (PF14639) and the SH2 (PF14633) domains are conserved in the *T. brucei* orthologue, as annotated by InterProScan5.

B. Reduction in Spt6 transcript in *T. brucei* RY-Spt6b after induction of Spt6 RNAi with tetracycline as analysed via qPCR. The relative change in transcript levels is plotted against time induction with tetracycline (Tet). The mean of three independent experiments is shown with the standard deviation indicated with error bars.

C. Spt6 is essential in bloodstream form *T. brucei.* The graph shows the growth of the *T. brucei* RY-Spt6a and RY-Spt6b cell lines in the presence (+) or absence (-) of tetracycline to induce Spt6 RNAi compared with the parental RY strain. The mean of three measurements is shown with the standard deviation indicated with error bars.

Sup. Fig. 3

Depletion of Spt16 disrupts higher order nucleosome structure, resulting in an increase in mononucleosomes at the silent VSG ES.

A. Ethidium bromide stained gels showing nucleosome fractions after digestion of genomic DNA (gDNA) with micrococcal nuclease (MNase) and fractionation over a sucrose gradient. *T. brucei* RY-Spt16 cells were grown for 24 hours in the absence (-) or presence (+) of tetracycline (Tet) to knockdown Spt16. An equal number of cells for both samples was subjected to MNase digestion, before separating the resulting nucleosome fractions by centrifugation over a sucrose gradient. DNA of each fraction was isolated by phenol/ chloroform extraction and subjected to gel electrophoresis. The total input (In) is shown, followed by fractions 8 to 24 (top to bottom). Fractions 1 to 7 contained very little DNA and are therefore not shown. For further analysis, fractions of RY-Spt16 (-Tet) or RY-Spt16 (+Tet) DNA containing similar species of nucleosomes were pooled as indicated into mono-, di-, di/tri-, tri/tetra-, and >tetranucleosome containing fractions.

B. Knockdown of Spt16 results in increased MNase sensitivity (increased abundance of mononucleosomes) specifically at the silent VSG ES promoter. The amount of each nucleosome-fraction (percentage total digested DNA) in RY-Spt16 cells (-Tet versus +Tet) as shown in Sup. Fig. 3A is shown. Quantitative PCR (qPCR) with primers specific to the active *VSG T3* or the silent *VSG 221* ES was used to determine the abundance of specific genomic loci within the respective fractions. In order to adjust for the different amount of gDNA derived from the same number of cells before and after knockdown of Spt16 (due to the G2/M cell cycle arrest), the percentage of each pooled fraction from total (Σ (mono + di + di/tri + tri/tetra + >tetra + bottom fraction) = 100%) in RY-Spt16 (-Tet) or (+Tet) was determined, before being plotted as percentage according to Povelones *et al* (2012). The results are the mean of three independent experiments, with the standard deviation indicated with error bars. Significance was determined by a Student's t-test (unpaired, two-tailed), and indicated with an asterisk (p=0.01-0.05). All obtained p-values are listed in Supplementary Table 2.

Sup. Fig. 4

Depletion of Spt16 does not significantly affect chromatin structure at Pol II and non-ES Pol I transcription units, showing only a trend to increasing tri-and tetra- nucleosomes.

The percentage of each nucleosome fraction in the total digested gDNA of RY-Spt16 cells (-Tet versus +Tet) as indicated in Sup. Fig. 3A is shown. Quantitative PCR (qPCR) with primers specific for silent and active Pol I transcription units (as illustrated in Fig. 3C), as well as Pol II transcription units, was used to determine the abundance of those genomic loci within the respective fractions. In order to adjust for the different amounts of gDNA obtained from the same number of cells before and after knockdown of Spt16 (due to the G2/M cell cycle arrest), the percentage of each pooled fraction from total (Σ (mono + di + di/tri + tri/tetra + >tetra + bottom fraction) = 100%) in RY-Spt16 (-Tet or +Tet) was determined, before being plotted as a percentage of total according to Povelones *et al* (2012). The results are the mean of three independent experiments, with the standard deviation indicated with error bars. Significance was determined by a Student's t-test (unpaired, two-tailed). All obtained p-values are listed in Supplementary Table 2.

Sup. Fig. 5

The FACT large subunit Spt16 is relatively depleted at the Pol I rDNA promoter, but is evenly distributed across the Pol II convergent and divergent strand switch regions (SSRs).

A. Schematic of a *T. brucei* rDNA transcription unit. The rRNA genes are shown as filled boxes and the promoter with a flag. The regions used for qPCR are indicated with lettered bars. The bar graph shows quantitation of ChIP experiments performed with a cell line containing myc-tagged Spt16 compared with the parental cell line (wild type or WT). The amount of Spt16 found binding to the rDNA locus was determined using qPCR. The mean of two independent experiments is shown.

B. The schematics show a representative Pol II convergent and divergent SSR (C2 and D1 of chromosome 10) with qPCR primers indicated with lettered dots as in (Stanne *et al.*, 2011).

The bar graphs show quantitation of the ChIP of myc-tagged Spt16 binding to various loci in comparison with a ChIP performed with the wild type strain, plotted as percentage of the total input. The mean of two independent experiments is shown.

Sup. Fig. 6

Depletion of Spt16 results in a reduction in rRNA precursor transcripts and an increase in histone abundance at the rDNA.

A. Schematic of a *T. brucei* rDNA transcription unit. The rRNA genes are shown as filled boxes, and the promoter with a flag. The regions used for qPCR analysis are indicated with lettered bars. Three rRNA precursor transcripts are indicated below as described in (White *et al.*, 1986).

B. Significant decrease in rRNA precursor transcripts from the 3' end of the rDNA transcription unit. The fold change in rRNA precursors as determined by qPCR is plotted through time after induction of Spt16 RNAi using tetracycline (Tet). Precursor 1 (pre 1) was detected with primer pair 'm', precursor 2 with primer pair 'n' and precursors 2 and 3 with primer pair 'p'. The mean of three independent experiments is shown with the standard deviation indicated with error bars. Significance was determined using a Student's t-test (unpaired, two-tailed) with significant: * (p=0.01-0.05) indicated.

C. Increase in histone H3 at the rDNA after depletion of the FACT large subunit Spt16. The bar graphs show the abundance of histones H3 and H2A as determined by ChIP and subsequent qPCR analysis using the primers indicated in panel A. The ChIP results were obtained in the presence (+) or absence (-) of Spt16 RNAi for 24 hours. The results are shown as the mean of three independent experiments with the standard deviation indicated with error bars. The statistical significance was determined using a Student's t-test (unpaired, two-tailed).

D. No significant change in histone H3 and H2A distribution after knockdown of the putative histone chaperone Spt6. Further the experiments in this panel are as described in panel C.

Sup. Fig. 7

Decrease in Pol II precursor transcripts after depletion of Spt16.

A. Schematic of the *T. brucei* tubulin array comprising alternating α- and β- tubulin genes. The intergenic regions used to assess levels of tubulin precursor transcript are indicated with black bars.

B. Decrease in tubulin precursor transcript (α/ β pre) after induction of Spt16 RNAi with tetracycline (Tet) for the time indicated. The mean of three independent experiments is shown with the standard deviation indicated with error bars. Statistical significance was determined with significant: * (p=0.01-0.05) indicated.

Sup. Fig. 8

There is no significant change in levels of histone H3 or H2A at or around Pol II transcription units after depletion of Spt6.

The relative abundance of histones H3 and H2A was determined at Pol II transcription units and strand switch regions (SSR) after Spt6 knockdown. Convergent regions contain putative Pol II terminators and divergent regions putative promoters. Histone ChIP material was analysed by qPCR. The schematics show Pol II convergent and divergent SSRs (C2 and D1 of chromosome 10) with qPCR primers used for analysis indicated with lettered dots as in (Stanne et al., 2011). The bar graphs show histone H3 or H2A levels in the *T. brucei* RY-Spt6 strain in the presence (+) or absence (-) of tetracycline for 24 hours to induce Spt6 RNAi. The Pol II transcription units analysed include the large subunit of RNA

polymerase I (Pol I), α-tubulin (tub), γ-tubulin and actin, as well as the Pol II transcription units around the depicted SSRs. Results are the mean of three independent experiments, with the standard deviation indicated with error bars. Statistical significance was determined with significant: * (p=0.01-0.05) indicated.

Sup. Fig. 9

Depletion of Cyclin 6 is lethal in bloodstream form *T. brucei* and results in a cell cycle arrest with an accumulation of 1N2K cells.

A. Cyclin 6 is essential in bloodstream form *T. brucei*. The growth of the *T. brucei* RY-Cyc6a and RY-Cyc6b strains in the presence (+) or absence (-) of tetracycline to induce Cyc6 RNAi is compared with the parental RY strain. The mean of three measurements is shown with the standard deviation indicated with error bars.

B. Cell cycle analysis using the DNA stain DAPI after the induction of Cyc6 RNAi with tetracycline (Tet) for the time in hours (h) indicated. The percentage of cells (n~300) with different numbers of nuclei (N) or kinetoplasts (K) is indicated. The mean of three independent experiments is shown with the standard deviation indicated with error bars.

C. Reduction in cyclin 6 transcript as determined by qPCR after the induction of Cyc6 RNAi using tetracycline (Tet) for the indicated time.

Sup. Fig. 10

Depletion of Cyclin 6 as well as Spt16 results in cell cycle arrest at G2/ M.

A. Accumulation of cells in the G2/ M cell cycle phase (4n DNA content) after induction of Cyc6 RNAi using tetracycline (Tet). Cells were stained with propidium iodide, and DNA content determined in the FL2 channel using flow cytometry (representative experiment shown). The relevant gates used for quantitation are indicated with brackets. The graph

shows the mean of three independent experiments with standard deviation indicated with error bars.

B. Knockdown of Spt16 also results in an accumulation of cells in the G2/ M cell cycle stage. The experiments in this panel are further as described above.
